# Annual report on National Clinical Database 2020 for gastroenterological surgery in Japan

**DOI:** 10.1002/ags3.12662

**Published:** 2023-02-09

**Authors:** Yoshiki Kajiwara, Arata Takahashi, Hideki Ueno, Yoshihiro Kakeji, Hiroshi Hasegawa, Susumu Eguchi, Takanori Goi, Akio Saiura, Akira Sasaki, Shuji Takiguchi, Hiroya Takeuchi, Chie Tanaka, Masaji Hashimoto, Naoki Hiki, Akihiko Horiguchi, Satoru Matsuda, Tsunekazu Mizushima, Shigeru Marubashi, Mitsukazu Gotoh, Hiroyuki Konno, Hiroyuki Yamamoto, Hiroaki Miyata, Yasuyuki Seto, Yuko Kitagawa, The National Clinical Database

**Affiliations:** ^1^ The Japanese Society of Gastroenterological Surgery Tokyo Japan; ^2^ Department of Health Policy and Management, School of Medicine Keio University Tokyo Japan; ^3^ Department of Healthcare Quality Assessment Graduate School of Medicine The University of Tokyo Tokyo Japan

**Keywords:** annual report, gastroenterological surgery, National Clinical Database, short‐term outcome, surgical outcome

## Abstract

**Aim:**

The National Clinical Database (NCD) of Japan is a nationwide data entry system for surgery, and it marked its 10th anniversary in 2020. The aim was to present the 2020 annual report of gastroenterological surgery of the NCD.

**Methods:**

The data of the surgical procedures stipulated by the training curriculum for board‐certified surgeons of the Japanese Society of Gastroenterological Surgery in the NCD from 2011 to 2020 were summarized.

**Results:**

In total, 5 622 845 cases, including 593 088 cases in 2020, were extracted from the NCD. The total number of gastroenterological surgeries increased gradually in these 10 years, except for the year 2020 due to the COVID‐19 pandemic. The annual number of surgeries of each organ, except the pancreas and liver, decreased by 0.4%–13.1% in 2020 compared to 2019. The surgical patients were consistently aging, with more than 20% of all gastroenterological surgeries in 2020 involving patients aged 80 years or older. The participation of board‐certified surgeons increased for each organ (75.9%–95.7% in 2020). The rates of endoscopic surgery also increased constantly. Although the incidences of postoperative complications of each organ increased by 0.7%–7.9% in these 10 years, postoperative mortality rates decreased by 0.2%–1.5%.

**Conclusions:**

We present here the short‐term outcomes of each gastroenterological operative procedure in 2020. This review of the 10‐years of NCD data of gastroenterological surgery revealed a consistent increase of the number of surgeries (except for in 2020), especially endoscopic procedures, and aging of the Japanese population. The good safety of Japanese gastroenterological surgeries was also indicated.

## Graphical abstract

This study presents the annual report on the National Clinical Database 2020 for gastroenterological surgery in Japan. This manuscript revealed a consistent increase of the number of surgeries, especially with endoscopic surgeries, and aging of the Japanese population. Moreover, it indicated the good safety of Japanese gastroenterological surgeries, most of which have been increasingly carried out by the board‐certified surgeons.

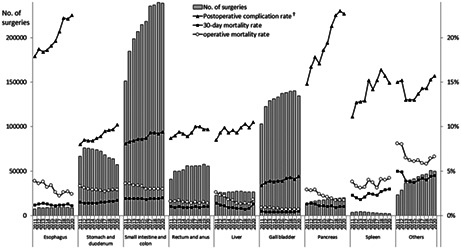

## 1 INTRODUCTION

The Japanese National Clinical Database (NCD) is a large‐scale, nationwide, web‐based data entry system for surgical procedures that was established with major support from the many Japanese professional surgical societies, including the Japanese Society of Gastroenterological Surgery (JSGS), and it marked its 10th anniversary in 2020.^1^ The NCD has grown into a large, nationwide database covering more than 95% of the surgeries performed by general surgeons in Japan.^2^ The NCD started its data registration in 2011, and since then over 5500 facilities have enrolled, and over 14 000 000 cases have been registered in 10 years.^3^

The JSGS specified the 121 gastroenterological operative procedures as a requirement for board certification. Gastroenterological surgical procedures are classified into three groups according to their technical level: low, medium, and high degree of difficulty. In addition, the JSGS specified the eight major procedures (esophagectomy, distal gastrectomy, total gastrectomy, right hemicolectomy, low anterior resection, hepatectomy, pancreaticoduodenectomy, and acute diffuse peritonitis surgery) for special emphasis in terms of medical standards for improvement of surgical quality.^4^ All surgical cases were registered in the NCD with the data for postoperative short‐term outcomes and detailed data, such as comorbidities and morbidities, for the eight major procedures. Risk models of mortality^5^, ^6^, ^7^, ^8^, ^9^, ^10^, ^11^, ^12^ and morbidity^13^, ^14^, ^15^, ^16^, ^17^, ^18^, ^19^, ^20^, ^21^ for these eight major procedures have been reported, which were the risk stratification studies based on all real‐world Japanese data from the NCD. Based on these studies, a real‐time feedback system, which includes a risk calculator for the expected mortalities and morbidities of preoperative patients, was established on the NCD website.^4^ This feedback system shows each facility's severity‐adjusted clinical performance compared to the national data. It also indicates the risk‐adjusted cumulative expected and observed number of deaths in individual institutions.

The major purpose of the NCD is to contribute to the maintenance or improvement of medical quality. Therefore, the data registered in the NCD have been used steadily, as mentioned above. Data reliability and disclosure are also vitally important. For the former, to assure the reliability of data of gastroenterological surgeries that had been collected by the NCD, the JSGS started data verification annually in 2015 and demonstrated the high accuracy of the accumulated data.^22^, ^23^ As for the latter, NCD annual reports, which reflect the real‐world surgical outcomes of Japanese gastroenterological surgeries, have been published.^24^, ^25^, ^26^, ^27^ In this paper, following previous annual reports, the short‐term outcomes of each gastroenterological operative procedure in 2020 will be reported. Additionally, important findings on the changes over time in gastroenterological surgeries in Japan between 2011 and 2020 will be described.

## 2 SUBJECTS AND METHODS

The subjects were patients who underwent one or more of the 121 surgical procedures specified by the Training Curriculum for Board‐Certified Surgeons in Gastroenterology and had their surgical data recorded from 2011 to 2020 in the NCD system. As previously reported,^24^, ^25^, ^26^, ^27^ the clinical data of these patients were collected from the NCD database. Data were extracted in a secure system without any external connection, and basic statistical analysis was carried out by NCD statisticians. For each surgical procedure, the mortality rate and the number of surgical cases by sex, age, and postoperative complications were calculated. Postoperative complications that were grade III or more severe in the Clavien–Dindo (C‐D) classification^28^ were defined as severe complications. The proportion of the institutions certified by the JSGS and participation of specialists (anesthesiologists and board‐certified surgeons) were also calculated for each surgical procedure.

The training institutions (board‐certified institutions and their affiliated hospitals) and board‐certified surgeons in gastroenterology are stipulated by the JSGS. The major requirements for board‐certified institutions include (A) 600 or more gastroenterological operations stipulated by the certification committee, of which more than 120 are bound to be essential major surgeries, in the last 3 years, (B) one or more consultant surgeons (board‐certified surgeons who are certified as educators by the JSGS based on the clinical and academic experiences), and (C) sufficient educational opportunities and academic activity. For affiliated institutions of board‐certified institutions, at least one board‐certified surgeon, 20 beds for gastroenterological division, and specified curriculum systems are required. Board‐certified surgeons need the experience of 300 or more gastroenterological operations and gastroenterological surgical training for more than 4 years according to the training curriculum in training institutions of the JSGS as noted above.

The following points need to be considered in the interpretation of the data reported here. First, cases in which several operative methods were performed simultaneously were tallied per individual operative methods. Since a maximum of eight operative procedures can be recorded per case in the NCD, the total number of surgeries for each result does not represent the actual total number of surgical cases. Second, cases with errors in patient age, sex, and postoperative 30‐day status were excluded. Thus, four surgical procedures (hemorrhoidectomy, perianal abscess drainage, surgery for anal fistula, and abdominal/inguinal hernioplasty) were excluded in this report, because postoperative 30‐day status was unknown. Finally, postoperative 30‐day mortality included all cases of death within 30 days from surgery, whether or not the patient was discharged. Operative mortality was a number that combined 30‐day mortality and in‐hospital deaths 31–90 days after surgery.

## 3 RESULTS

### 3.1 Gastroenterological operative procedures in the “Training Curriculum for Board‐Certified Surgeons in Gastroenterology” in 2020

The total number of patients who underwent gastroenterological surgeries recorded in the NCD from January 1 to December 31 in 2020 was 593 088. In the analyses regarding the treated organ, 8713 cases included the esophagus (1.5%); 57 171 cases, the stomach and duodenum (9.6%); 238 631 cases, the small intestine and colon (40.2%); 55 536 cases, the rectum and anus (9.3%); 26 614 cases the liver (4.5%); 134 332 cases, the gallbladder (22.6%); 19 947 cases, the pancreas (3.4%); 2096 cases, the spleen (0.4%); and 50 048 cases, other organs (8.4%).

Table 1 shows the number of surgeries and characteristics of each operative procedure in the “Training Curriculum for Board‐Certified Surgeons in Gastroenterology” in 2020. The percentage of surgeries performed with the participation of an anesthesiologist was approximately 95%, except for anal surgeries (e.g., anal sphincteroplasty, 21.5%; transanal rectal tumor resection, 36.0%; proctocele surgery, 55.2%). The rates of participation of JSGS board‐certified surgeons in 2020 were approximately 95% for the surgeries of esophagus, liver, and pancreas. In contrast, the rates were relatively low for surgeries of the small intestine and colon (mean: 75.9%), gallbladder (77.7%), and other organs (76.1%).

**TABLE 1:** Characteristics of each operative procedures of the “Training Curriculum for Board‐Certified Surgeons in Gastroenterology” in 2020

Organ	Difficulty level	Operative procedure	No. of surgeries	Sex male (%)	Age ≥ 80 (%)	Anesthesiologist participation (%)	Board‐certified surgeon participation (%)	Operating surgeon (%)	
								Board‐certified surgeon	Non‐board‐ certified surgeon
Esophagus	Low	Cervical periesophageal abscess drainage	34	79.4	14.7	97.1	94.1	70.6	29.4
	Med	Esophageal suture (perforation, injury)	178	78.1	20.2	94.9	87.1	59.0	41.0
	Med	Thoracic periesophageal abscess drainage	18	94.4	16.7	100.0	94.4	83.3	16.7
	Med	Esophageal foreign body extraction	30	63.3	23.3	96.7	86.7	70.0	30.0
	Med	Esophageal diverticulum resection	40	57.5	12.5	100.0	85.0	77.5	22.5
	Med	Benign esophageal tumor removal	44	40.9	0.0	100.0	100.0	84.1	15.9
	Med	Esophageal resection (removal only)	607	80.4	14.2	98.4	91.6	72.3	27.7
	Med	Esophageal reconstruction (gastric tube reconstruction)	535	80.0	7.7	98.9	96.4	79.4	20.6
	Med	Esophageal fistula construction	172	87.2	12.8	94.2	94.8	83.1	16.9
	Med	Esophagocardioplasty	218	43.1	30.3	95.0	80.3	54.6	45.4
	Med	Achalasia surgery	189	54.0	10.1	97.4	57.7	32.3	67.7
	High	Esophagectomy	6111	80.4	8.2	99.3	98.0	82.1	17.9
	High	Esophageal reconstruction (colon reconstruction)	21	90.5	19.0	100.0	95.2	85.7	14.3
	High	Esophageal bypass	135	85.9	9.6	99.3	94.8	58.5	41.5
	High	Bronchoesophageal fistula surgery	7	57.1	0.0	100.0	100.0	42.9	57.1
	High	Secondary esophageal reconstruction	374	88.2	11.2	99.2	98.1	75.4	24.6
Stomach and duodenum	Low	Gastrostomy and suture gastrorrhaphy	69	46.4	15.9	94.2	73.9	27.5	72.5
	Low	Diverticulectomy, polypectomy (excluding endoscopic resection)	162	51.2	16.0	95.1	88.9	54.9	45.1
	Low	Truncal vagotomy	1	100.0	0.0	100.0	100.0	0.0	100.0
	Low	Gastroenterostomy (Including duodenal jejunostomy)	5908	62.5	27.7	95.4	83.5	38.8	61.2
	Low	Gastric fistula construction (Excluding PEG)	1511	68.0	29.3	92.1	76.2	40.9	59.1
	Low	Gastric pyloroplasty	109	75.2	6.4	95.4	66.1	31.2	68.8
	Low	Gastric volvulus surgery and rectopexy	60	45.0	38.3	95.0	76.7	40.0	60.0
	Low	Gastric suture (including gastric suture for gastric rupture, suture closure for gastroduodenal perforation, omental implantation and omental transposition)	5246	65.5	24.6	93.2	72.1	28.7	71.3
	Low	Local gastrectomy (including wedge resection)	4504	49.6	13.9	96.6	88.6	49.5	50.5
	Med	Gastrectomy (including distal, pylorus preserving and segmental)	28 944	67.1	25.4	96.1	87.9	51.4	48.6
	Med	Selective vagotomy	2	100.0	0.0	100.0	100.0	50.0	50.0
	High	Total gastrectomy (including proximal gastrectomy)	10 652	75.2	22.0	96.0	86.7	51.8	48.2
	High	Left upper abdominal exenteration	3	66.7	0.0	100.0	100.0	100.0	0.0
Small intestine and colon	Low	Enterotomy and enterorrhaphy	4477	57.4	23.9	93.3	78.2	38.1	61.9
	Low	Disinvagination (invasive)	168	56.5	25.0	94.0	69.0	22.0	78.0
	Low	Partial small bowel resection (benign)	9046	57.0	30.6	94.6	76.0	33.2	66.8
	Low	Ileocecal resection (benign)	4783	57.1	17.7	94.4	76.6	31.5	68.5
	Low	Partial colectomy and sigmoid colectomy (benign)	8204	61.0	26.7	94.4	76.8	32.8	67.2
	Low	Appendectomy	57 282	55.7	6.0	92.2	63.8	21.7	78.3
	Low	Enterostomy and closure (without enterectomy)	27 445	63.2	20.0	95.4	79.9	41.6	58.4
	Med	Small bowel resection (malignant)	3853	54.5	18.8	96.4	80.6	40.3	59.7
	Med	Ileocecal resection (malignant)	14 587	46.0	32.2	96.0	82.2	34.6	65.4
	Med	Partial colectomy and sigmoid colectomy (malignant)	30 372	57.1	24.1	96.1	84.3	43.4	56.6
	Med	Right hemicolectomy	21 626	51.1	32.9	95.4	81.5	40.3	59.7
	Med	Left hemicolectomy	6037	58.1	23.8	95.8	80.9	44.2	55.8
	Med	Total colectomy	1565	58.8	22.3	96.1	81.1	45.4	54.6
	Med	Intestinal obstruction surgery (with enterectomy)	25 480	49.8	37.5	93.8	73.4	30.7	69.3
	Med	Enterostomy and closure (with enterectomy)	23 316	63.4	18.0	95.5	79.7	38.2	61.8
	High	Proctocolectomy and ileoanal (canal) anastomosis	390	64.4	1.3	96.4	95.4	67.9	32.1
Rectum	Low	Transanal rectal tumor resection	3469	49.9	13.2	36.0	58.0	38.7	61.3
	Low	Proctocele surgery (transanal)	2330	14.4	63.6	55.2	59.5	34.1	65.9
	Med	Abdominoperineal resection (benign)	1120	60.5	18.8	95.0	81.4	49.6	50.4
	Med	High anterior resection	11 276	58.8	18.3	96.1	86.5	48.4	51.6
	Med	Hartmann's procedure	6518	54.8	38.2	95.1	79.9	35.5	64.5
	Med	Proctocele surgery (abdominoperineal)	1983	10.9	59.1	90.7	76.6	46.4	53.6
	Med	Malignant anorectal tumor excision (transanal)	707	53.7	29.1	81.0	78.5	52.1	47.9
	Med	Anal sphincteroplasty (by tissue replacement)	2591	53.5	11.3	21.5	56.1	40.4	59.6
	High	Abdominoperineal resection (malignant)	4959	63.4	22.1	96.5	88.6	60.3	39.7
	High	Low anterior resection	20 022	63.9	13.7	96.1	88.2	60.6	39.4
	High	Total pelvic exenteration	517	68.1	5.2	97.7	91.5	73.9	26.1
	High	Anorectal malignant tumor excision (posterior approach)	44	47.7	11.4	88.6	79.5	45.5	54.5
Liver	Low	Hepatorrhaphy	70	68.6	17.1	88.6	72.9	37.1	62.9
	Low	Liver abscess drainage (excluding percutaneous procedures)	52	57.7	44.2	96.2	84.6	38.5	61.5
	Low	Hepatic cyst resection, suture, drainage	900	26.4	19.8	96.2	87.9	47.3	52.7
	Low	Liver biopsy (excluding percutaneous procedures)	291	52.9	4.8	82.5	84.9	38.8	61.2
	Low	Liver coagulo‐necrotic therapy (excluding percutaneous procedures)	534	74.3	19.1	97.9	94.4	65.7	34.3
	Med	Partial hepatectomy	12 668	66.7	14.2	98.0	94.8	65.3	34.7
	Med	Lateral segmentectomy	1451	65.8	18.7	97.6	93.3	60.6	39.4
	Med	Esophageal and gastric varix surgery	44	54.5	4.5	68.2	38.6	20.5	79.5
	High	Surgery for hepatic trauma (excluding drainage only)	363	73.6	14.9	79.6	63.4	35.0	65.0
	High	Hepatectomy (segmentectomy or more; excluding lateral segmentectomy)	6753	68.4	14.7	98.1	97.3	76.5	23.5
	High	Subsegmentectomy	2664	71.7	14.1	97.4	96.8	74.2	25.8
	High	Liver transplantation	692	52.7	0.0	98.8	98.6	78.8	21.2
	High	Hepatopancreatoduodenectomy	132	77.3	7.6	98.5	99.2	81.1	18.9
Gall bladder	Low	Cholangiotomy	78	56.4	43.6	93.6	88.5	44.9	55.1
	Low	Cysticolithectomy	74	58.1	32.4	89.2	75.7	28.4	71.6
	Low	Cholecystectomy	127 454	55.8	17.2	94.7	77.1	32.7	67.3
	Low	External cholecystostomy	104	61.5	43.3	70.2	78.8	48.1	51.9
	Low	Cystoenteric anastomosis	37	51.4	45.9	97.3	73.0	40.5	59.5
	Med	Cysticolithectomy	2360	60.5	40.5	93.6	82.1	40.6	59.4
	Med	Biliary tract reconstruction	325	53.2	18.2	98.8	95.1	69.8	30.2
	Med	Biliary bypass	1112	57.0	25.6	95.8	91.1	53.7	46.3
	Med	Cholangioplasty	114	57.0	16.7	96.5	93.9	73.7	26.3
	Med	Duodenal papilloplasty	15	66.7	26.7	100.0	80.0	46.7	53.3
	Med	Choledocal dilatation	265	22.6	1.1	98.5	88.3	55.8	44.2
	Med	Biliary fistula closure	35	69.7	15.2	97.0	75.8	36.4	63.6
	High	Surgery for bile duct trauma (excluding drainage only)	225	58.2	25.3	96.9	83.6	45.8	54.2
	High	Malignant gallbladder tumor surgery (excluding simple cholecystectomy)	1019	55.4	23.6	97.4	93.4	57.5	42.5
	High	Malignant bile duct tumor surgery	1093	68.5	20.9	97.3	96.7	76.9	23.1
	High	Biliary atresia surgery	24	16.7	4.2	100.0	58.3	16.7	83.3
Pancreas	Low	External pancreatic cyst drainage	2	0.0	0.0	100.0	50.0	0.0	100.0
	Low	External pancreatic duct drainage	12	66.7	8.3	75.0	83.3	58.3	41.7
	Med	Pancreatorrhaphy	6	33.3	0.0	100.0	83.3	50.0	50.0
	Med	Partial pancreatic resection	174	45.4	10.9	97.7	93.1	73.6	26.4
	Med	Distal pancreatectomy (benign)	1401	43.3	9.0	96.7	94.8	69.7	30.3
	Med	Pancreatic cyst‐enterostomy	27	77.8	18.5	96.3	96.3	59.3	40.7
	Med	Pancreatic (duct) enterostomy	289	68.2	7.6	94.5	96.9	74.7	25.3
	Med	Acute pancreatitis surgery	78	80.8	19.2	73.1	64.1	38.5	61.5
	Med	Pancreatolithiasis surgery	27	81.5	0.0	96.3	88.9	63.0	37.0
	Med	Plexus pancreaticus capitalis resection	0	‐	‐	‐	‐	‐	‐
	High	Surgery for pancreatic trauma (excluding drainage only)	54	70.4	11.1	90.7	77.8	42.6	57.4
	High	Pancreaticoduodenectomy	11 953	60.6	16.0	97.9	96.4	71.2	28.8
	High	Distal pancreatectomy (malignant)	4912	56.2	18.0	97.7	94.8	68.5	31.5
	High	Total pancreatectomy	636	0.0	0.0	100.0	100.0	100.0	0.0
	High	Duodenum preserving pancreas head resection	34	57.7	11.6	98.3	98.1	75.0	25.0
	High	Segmental pancreatic resection	157	61.8	14.7	97.1	100.0	70.6	29.4
	High	Pancreatic transplantation	20	49.7	5.1	98.1	94.3	72.0	28.0
Spleen	Low	Splenorrhaphy	36	66.7	19.4	94.4	69.4	33.3	66.7
	Med	Splenectomy	2045	54.3	12.3	96.5	88.7	56.1	43.9
	Med	Partial splenectomy	15	33.3	6.7	100.0	86.7	53.3	46.7
Other	Low	Localized intra‐abdominal abscess surgery	2376	60.1	18.0	93.1	73.4	33.5	66.5
	Low	Exploratory laparotomy	12 401	60.3	18.0	94.3	78.5	40.4	59.6
	Med	Acute diffuse peritonitis surgery	15 542	58.9	27.8	94.6	76.1	31.7	68.3
	Med	Ventral hernia surgery	14 136	46.4	21.6	93.6	71.8	35.2	64.8
	Med	Diaphragm suture	294	56.1	18.7	94.2	81.3	44.6	55.4
	Med	Esophageal hiatus hernia surgery	1217	33.5	39.6	96.1	87.9	56.0	44.0
	Med	Retroperitoneal tumor surgery	1551	48.2	9.4	96.6	85.1	58.3	41.7
	Med	Abdominal wall/mesenteric/omental tumor resection	2054	50.1	11.0	96.4	80.6	44.4	55.6
	Med	Gastrointestinal perforation closure	421	64.1	29.5	90.0	75.3	35.4	64.6
	High	Diaphragmatic hiatus hernia surgery	56	44.6	25.0	96.4	87.5	53.6	46.4

The short‐term outcomes of each operative procedure are presented in Table 2. Among the 121 gastroenterological procedures that had been performed in over 100 cases in 2020, those with high operative mortality (>10%) were surgeries for hepatic trauma (excluding drainage only) (27.5%), gastrointestinal perforation closure (16.2%), hepatopancreatoduodenectomy (13.6%), total colectomy (13.5%), external cholecystostomy (13.5%), esophageal fistula construction (12.8%), acute diffuse peritonitis surgery (11.5%), and partial small bowel resection (for benign) (10.6%). Of these eight procedures, the difficulty level was high in two procedures (surgery for hepatic trauma and hepatopancreatoduodenectomy) and moderate or low in the others.

**TABLE 2:** Number of surgeries and short‐term outcome of each operative procedures of the “Training Curriculum for Board‐Certified Surgeons in Gastroenterology” in 2020

Organ	Difficulty level	Operative procedure	No. of surgeries	Endoscopic surgeries (%)	Emergency surgeries (%)	Postoperative complications^a^ (%)	re‐operations (%)	Postoperative 30‐day mortalities (%)	Operative mortalities^b^ (%)
Esophagus	Low	Cervical periesophageal abscess drainage	34	17.6	73.5	29.4	8.8	2.9	8.8
	Med	Esophageal suture (perforation, injury)	178	11.8	70.8	35.4	12.9	4.5	9.0
	Med	Thoracic periesophageal abscess drainage	18	11.1	100.0	50.0	27.8	0.0	0.0
	Med	Esophageal foreign body extraction	30	16.7	76.7	13.3	10.0	0.0	3.3
	Med	Esophageal diverticulum resection	40	22.5	5.0	7.5	7.5	0.0	0.0
	Med	Benign esophageal tumor removal	44	77.3	0.0	6.8	2.3	0.0	0.0
	Med	Esophageal resection (removal only)	607	60.1	6.3	16.6	14.8	1.5	4.6
	Med	Esophageal reconstruction (gastric tube reconstruction)	535	67.1	0.4	20.2	4.7	1.1	3.4
	Med	Esophageal fistula construction	172	46.5	27.9	33.1	29.1	5.2	12.8
	Med	Esophagocardioplasty	218	72.5	3.2	11.0	5.5	1.4	1.8
	Med	Achalasia surgery	189	86.8	0.5	2.1	1.1	0.5	0.5
	High	Esophagectomy	6111	71.3	73.5	23.0	8.8	0.8	1.5
	High	Esophageal reconstruction (colon reconstruction)	21	47.6	70.8	28.6	12.9	0.0	0.0
	High	Esophageal bypass	135	17.8	100.0	31.1	27.8	3.7	8.9
	High	Bronchoesophageal fistula surgery	7	14.3	76.7	57.1	10.0	14.3	28.6
	High	Secondary esophageal reconstruction	374	12.0	5.0	32.6	7.5	1.3	3.5
Stomach and duodenum	Low	Gastrostomy and suture gastrorrhaphy	69	21.7	47.8	5.8	4.3	2.9	2.9
	Low	Diverticulectomy, polypectomy (excluding endoscopic resection)	162	12.3	13.0	7.4	3.1	0.6	0.6
	Low	Truncal vagotomy	1	100.0	0.0	0.0	0.0	0.0	0.0
	Low	Gastroenterostomy (including duodenal jejunostomy)	5908	29.5	9.2	15.9	4.4	4.8	8.9
	Low	Gastric fistula construction (excluding PEG)	1511	17.6	16.1	18.9	5.4	4.5	8.9
	Low	Gastric pyloroplasty	109	20.2	41.3	4.6	1.8	0.0	0.9
	Low	Gastric volvulus surgery and rectopexy	60	58.3	23.3	5.0	1.7	3.3	3.3
	Low	Gastric suture (including gastric suture for gastric rupture, Suture closure for gastroduodenal perforation, omental implantation and omental transposition)	5246	35.0	89.8	18.1	5.1	4.4	7.0
	Low	Local gastrectomy (including wedge resection)	4504	69.3	3.0	3.2	1.5	0.3	0.6
	Med	Gastrectomy (including distal, pylorus preserving and segmental)	28 944	53.8	1.9	7.7	2.5	0.8	1.3
	Med	Selective vagotomy	2	50.0	50.0	0.0	0.0	0.0	0.0
	High	Total gastrectomy (including proximal gastrectomy)	10 652	30.4	1.9	12.0	4.0	1.3	2.2
	High	Left upper abdominal exenteration	3	66.7	0.0	33.3	0.0	0.0	33.3
Small intestine and colon	Low	Enterotomy and enterorrhaphy	4477	16.8	29.7	18.1	7.6	4.5	8.4
	Low	Disinvagination (invasive)	168	27.4	81.5	11.9	4.8	5.4	6.5
	Low	Partial small bowel resection (benign)	9046	19.0	63.3	21.8	10.1	7.3	10.2
	Low	Ileocecal resection (benign)	4783	43.8	48.3	9.6	3.8	2.3	2.8
	Low	Partial colectomy and sigmoid colectomy (benign)	8204	33.3	46.6	16.0	6.3	4.1	5.6
	Low	Appendectomy	57 282	70.8	69.2	1.8	0.9	0.1	0.2
	Low	Enterostomy and closure (without enterectomy)	27 445	37.0	28.5	17.3	7.7	4.0	6.6
	Med	Small bowel resection (malignant)	3853	29.2	18.1	11.9	5.2	2.4	3.9
	Med	Ileocecal resection (malignant)	14 587	62.5	5.3	5.2	2.1	0.6	1.1
	Med	Partial colectomy and sigmoid colectomy (malignant)	30 372	61.9	3.7	6.7	3.5	0.6	1.0
	Med	Right hemicolectomy	21 626	54.2	8.5	8.0	3.3	1.4	2.2
	Med	Left hemicolectomy	6037	54.2	9.6	10.2	5.5	2.0	2.7
	Med	Total colectomy	1565	30.1	36.9	26.5	8.9	10.6	13.5
	Med	Intestinal obstruction surgery (with enterectomy)	25 480	23.7	66.6	10.3	4.2	2.4	3.4
	Med	Enterostomy and closure (with enterectomy)	23 316	17.3	21.2	14.0	5.1	3.2	4.8
	High	Proctocolectomy and ileoanal (canal) anastomosis	390	55.6	5.4	13.8	7.7	0.0	0.3
Rectum	Low	Transanal rectal tumor resection	3469	2.8	2.0	0.4	0.4	0.0	0.1
	Low	Proctocele surgery (transanal)	2330	0.4	0.9	1.5	1.9	0.3	0.5
	Med	Abdominoperineal resection (benign)	1120	12.1	18.8	17.9	6.3	1.9	2.9
	Med	High anterior resection	11 276	71.4	3.7	6.2	3.3	0.5	0.7
	Med	Hartmann's procedure	6518	21.2	57.4	20.7	5.7	5.4	7.3
	Med	Proctocele surgery (abdominoperineal)	1983	57.2	0.7	1.9	1.7	0.2	0.2
	Med	Malignant anorectal tumor excision (transanal)	707	13.4	6.4	5.8	5.0	1.0	1.6
	Med	Anal sphincteroplasty (by tissue replacement)	2591	1.7	2.2	1.1	1.3	0.1	0.2
	High	Abdominoperineal resection (malignant)	4959	70.9	1.0	12.3	4.6	0.6	1.1
	High	Low anterior resection	20 022	72.9	1.4	11.1	6.7	0.3	0.5
	High	Total pelvic exenteration	517	26.3	0.6	26.3	8.5	0.8	2.1
	High	Anorectal malignant tumor excision (posterior approach)	44	6.8	2.3	2.3	2.3	0.0	0.0
Liver	Low	Hepatorrhaphy	70	8.6	87.1	28.6	18.6	12.9	15.7
	Low	Liver abscess drainage (excluding percutaneous procedures)	52	25.0	42.3	17.3	7.7	3.8	3.8
	Low	Hepatic cyst resection, Suture, Drainage	900	75.7	4.6	4.8	1.0	0.3	0.3
	Low	Liver biopsy (excluding percutaneous procedures)	291	12.0	19.6	5.2	5.5	1.4	2.1
	Low	Liver coagulo‐necrotic therapy (excluding percutaneous procedures)	534	20.8	0.4	5.8	2.1	0.4	0.4
	Med	Partial hepatectomy	12 668	40.3	0.7	7.1	2.0	0.5	0.7
	Med	Lateral segmentectomy	1451	36.2	0.6	5.1	1.0	0.8	1.0
	Med	Esophageal and gastric varix surgery	44	47.7	22.7	11.4	2.3	2.3	4.5
	High	Surgery for hepatic trauma (excluding drainage only)	363	5.8	83.2	49.3	43.8	25.6	27.5
	High	Hepatectomy (segmentectomy or more; excluding lateral segmentectomy)	6753	15.5	0.3	15.2	3.0	1.4	2.3
	High	Subsegmentectomy	2664	28.4	0.3	9.0	1.5	0.5	0.9
	High	Liver transplantation	692	1.0	11.7	25.7	11.3	5.5	7.4
	High	Hepatopancreatoduodenectomy	132	0.0	0.8	60.6	7.6	8.3	13.6
Gall bladder	Low	Cholangiotomy	78	6.4	23.1	17.9	6.4	2.6	3.8
	Low	Cysticolithectomy	74	9.5	13.5	13.5	1.4	1.4	2.7
	Low	Cholecystectomy	127 454	70.9	15.7	3.7	1.1	0.4	0.6
	Low	External cholecystostomy	104	8.7	56.7	25.0	17.3	9.6	13.5
	Low	Cystoenteric anastomosis	37	8.1	13.5	8.1	2.7	5.4	5.4
	Med	Cysticolithectomy	2360	31.8	13.1	9.4	2.4	1.3	2.2
	Med	Biliary tract reconstruction	325	5.2	4.6	21.2	4.0	1.8	3.1
	Med	Biliary bypass	1112	5.2	9.6	15.4	4.8	2.4	4.3
	Med	Cholangioplasty	114	7.9	9.6	27.2	6.1	0.9	1.8
	Med	Duodenal papilloplasty	15	0.0	6.7	13.3	13.3	0.0	0.0
	Med	Choledocal dilatation	265	29.1	2.6	9.8	4.2	0.0	0.0
	Med	Biliary fistula closure	35	18.2	45.5	15.2	6.1	0.0	3.0
	High	Surgery for bile duct trauma (excluding drainage only)	225	28.9	40.9	26.7	10.7	7.1	9.8
	High	Malignant gallbladder tumor surgery (excluding simple cholecystectomy)	1019	7.5	0.7	12.2	2.7	0.5	0.5
	High	Malignant bile duct tumor surgery	1093	1.1	0.5	36.1	7.5	4.8	6.9
	High	Biliary atresia surgery	24	41.7	33.3	8.3	4.2	0.0	0.0
Pancreas	Low	External pancreatic cyst drainage	2	0.0	50.0	0.0	50.0	0.0	0.0
	Low	External pancreatic duct drainage	12	16.7	50.0	58.3	8.3	0.0	8.3
	Med	Pancreatorrhaphy	6	0.0	83.3	0.0	16.7	0.0	0.0
	Med	Partial pancreatic resection	174	30.5	0.6	20.7	3.4	0.6	1.1
	Med	Distal pancreatectomy (benign)	1401	49.4	3.0	18.2	2.6	0.3	0.7
	Med	Pancreatic cyst‐enterostomy	27	7.4	3.7	7.4	3.7	0.0	0.0
	Med	Pancreatic (duct) enterostomy	289	0.7	9.7	21.5	3.5	2.1	2.8
	Med	Acute pancreatitis surgery	78	3.8	44.9	41.0	39.7	9.0	20.5
	Med	Pancreatolithiasis surgery	27	3.7	0.0	11.1	0.0	0.0	0.0
	Med	Plexus pancreaticus capitalis resection	0	‐	‐	‐	‐	‐	‐
	High	Surgery for pancreatic trauma (excluding drainage only)	54	3.7	75.9	48.1	27.8	9.3	16.7
	High	Pancreaticoduodenectomy	11 953	3.4	0.6	24.1	3.4	1.2	1.8
	High	Distal pancreatectomy (malignant)	4912	26.4	0.7	20.6	2.5	0.5	1.1
	High	Total pancreatectomy	636	1.4	3.5	14.3	4.1	2.7	3.9
	High	Duodenum preserving pancreas head resection	34	0.0	2.9	26.5	2.9	2.9	2.9
	High	Segmental pancreatic resection	157	8.9	0.6	34.4	2.5	0.6	1.3
	High	Pancreatic transplantation	20	0.0	55.0	65.0	40.0	0.0	5.0
Spleen	Low	Splenorrhaphy	36	25.0	66.7	33.3	22.2	11.1	19.4
	Med	Splenectomy	2045	29.8	13.1	14.7	5.8	2.9	3.9
	Med	Partial splenectomy	15	53.3	13.3	0.0	0.0	0.0	0.0
Other	Low	Localized intra‐abdominal abscess surgery	2376	32.7	70.5	14.6	6.6	2.2	3.2
	Low	Exploratory laparotomy	12 401	49.3	30.3	16.8	13.2	6.2	9.0
	Med	Acute diffuse peritonitis surgery	15 542	22.1	92.6	27.3	7.8	8.0	11.5
	Med	Ventral hernia surgery	14 136	32.0	11.4	3.9	2.0	0.6	0.9
	Med	Diaphragm suture	294	34.0	40.5	19.7	9.5	4.4	6.5
	Med	Esophageal hiatus hernia surgery	1217	62.8	7.9	8.0	4.1	1.4	2.2
	Med	Retroperitoneal tumor surgery	1551	10.9	2.1	9.2	3.5	0.3	0.5
	Med	Abdominal wall/mesenteric/omental tumor resection	2054	31.3	15.3	7.7	4.3	0.9	1.9
	Med	Gastrointestinal perforation closure	421	11.9	90.5	35.4	14.0	11.6	16.2
	High	Diaphragmatic hiatus hernia surgery	56	37.5	42.9	12.5	3.6	10.7	12.5

^a^ Complications were defined by Clavien–Dindo grade IIIa–V.

^b^ Operative mortality was a rate that combined 30‐day mortality and hospitalization death in 31–90 days after surgery.

### 3.2 Annual changes in surgeries of each organ

**FIGURE 1:**
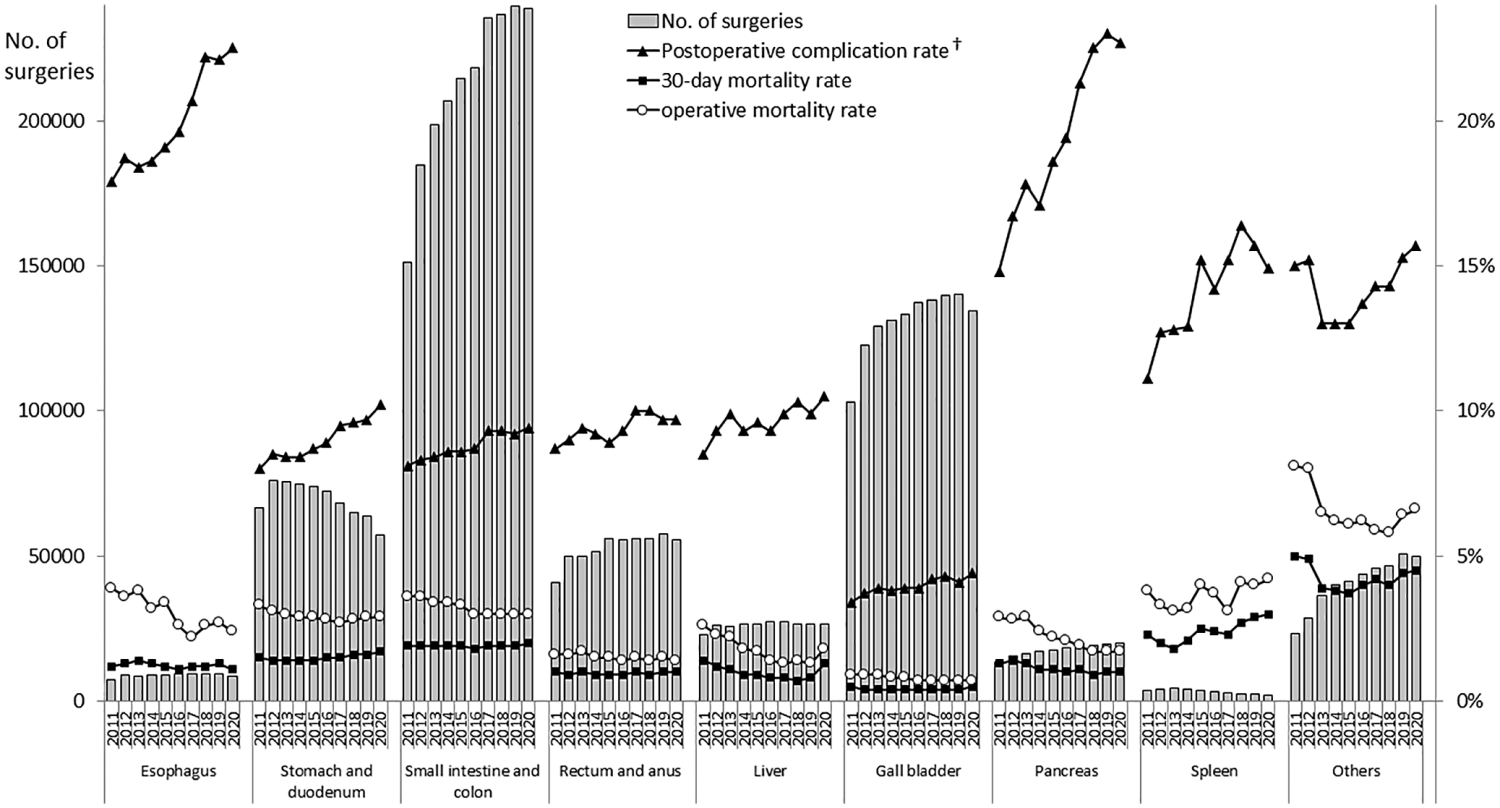
Annual changes in the number of surgeries, 30‐day and operative mortality rates, and complication rates of each organ. ^†^Postoperative complication was defined as grades IIIa–V in the Clavien–Dindo classification

The annual number of surgeries for each organ was decreased in 2020 compared to the prior year (esophagus, 5.5% decrease year‐on‐year; stomach and duodenum, 10.1%; small intestine and colon, 0.4%; rectum and anus, 3.8%; gallbladder, 4.2%; spleen, 13.1%; other organs; 0.9%), except for the pancreas (increase by 1.2% year‐on‐year) and liver (0.1%). The number of surgeries for the liver was almost stable, and the pancreas maintained the trend of slightly increasing through the 10 years (Figure 1).

The male‐to‐female ratio was approximately 8:2 for surgeries involving the esophagus, 7:3 for surgeries involving the liver and the stomach and duodenum, and 6:4 for surgeries involving other organs. For the spleen, the proportion of female increased by almost 7% in the last 10 years. The ratio of age ≥80 years increased 3.8%–6.1% in the last decade (Table 3). In terms of the institution types, most cases (93%–98% in 2020) for each organ were performed in certified or affiliated institutions of the JSGS (Table 4).

**TABLE 3:** Annual changes in surgeries of each organ by sex and age of patients

Organ	Year	No. of surgeries	Sex (%)		Age (%)					
			Male	Female	<60	60–64	65–69	70–74	75–79	≥80
Esophagus	2011	7246	81.8	18.2	22.5	19.6	21.1	18.7	12.0	6.0
	2012	8819	82.2	17.8	22.1	19.7	20.0	19.5	12.9	6.0
	2013	8642	81.5	18.5	20.8	17.5	21.0	20.6	13.2	6.9
	2014	9021	81.5	18.4	20.8	16.5	21.4	20.9	13.8	6.6
	2015	8943	80.8	19.2	19.6	15.3	22.4	22.5	13.1	7.1
	2016	9212	79.6	20.4	20.1	14.4	22.9	20.5	14.5	7.5
	2017	9359	80.0	20.0	19.3	13.4	24.4	19.4	15.5	8.0
	2018	9286	78.4	21.6	19.0	12.8	21.3	21.6	16.7	8.7
	2019	9224	78.6	21.4	18.8	13.1	19.4	22.8	17.3	8.6
	2020	8713	79.0	21.0	18.4	13.5	18.3	23.5	16.5	9.8
Stomach and duodenum	2011	66 740	68.0	32.0	20.1	14.4	14.0	17.1	16.4	18.0
	2012	76 186	68.3	31.7	18.9	14.4	14.5	17.1	16.4	18.6
	2013	75 583	67.9	32.1	18.6	13.1	15.5	17.2	16.9	18.7
	2014	74 920	67.6	32.4	17.9	12.1	16.0	17.8	16.7	19.5
	2015	73 877	67.8	32.2	17.4	11.1	17.1	17.8	16.6	19.9
	2016	72 234	67.8	32.2	17.0	10.2	18.1	17.1	16.6	21.0
	2017	68 287	67.2	32.8	16.3	9.9	17.5	17.3	17.2	21.8
	2018	65 152	66.9	33.1	16.0	9.0	16.4	18.0	17.5	23.2
	2019	63 610	66.5	33.5	15.6	8.8	15.0	19.0	18.5	23.2
	2020	57 171	66.6	33.4	15.3	8.2	13.8	20.1	18.5	24.1
Small intestine and colon	2011	151 143	56.7	43.3	37.4	10.9	10.5	12.1	12.2	16.9
	2012	184 810	56.7	43.3	36.4	10.7	10.7	12.2	12.5	17.4
	2013	198 677	56.9	43.1	35.6	10.1	11.3	12.7	12.4	17.8
	2014	206 857	56.9	43.1	34.7	9.4	12.0	13.1	12.4	18.4
	2015	214 453	57.1	42.9	34.0	8.9	12.9	13.1	12.3	18.7
	2016	218 228	57.3	42.7	33.7	8.4	13.6	12.5	12.4	19.3
	2017	235 359	56.7	43.3	32.7	8.0	13.2	12.7	12.9	20.5
	2018	236 496	56.9	43.1	32.2	7.7	12.6	13.4	13.2	21.1
	2019	239 612	56.3	43.7	32.1	7.4	11.7	13.9	13.5	21.2
	2020	238 631	56.2	43.8	32.6	7.3	10.7	14.6	13.4	21.5
Rectum and anus	2011	41 061	59.1	40.9	22.0	16.1	14.6	15.4	14.2	17.7
	2012	49 704	58.3	41.7	22.3	14.8	14.6	15.5	14.3	18.5
	2013	49 980	58.0	42.0	20.9	13.9	15.2	16.1	14.6	19.3
	2014	51 454	58.3	41.7	20.4	13.1	16.0	16.4	14.2	19.9
	2015	56 092	57.8	42.2	22.3	11.8	16.7	15.7	14.0	19.4
	2016	55 666	57.3	42.7	22.0	11.1	17.9	15.0	13.6	20.4
	2017	56 144	56.7	43.3	22.2	10.2	17.3	15.1	14.2	21.0
	2018	56 162	56.9	43.1	22.2	9.8	15.9	15.8	14.6	21.6
	2019	57 706	56.3	43.7	22.5	9.5	14.8	16.5	14.9	21.9
	2020	55 536	56.2	43.8	22.7	9.2	13.7	17.6	14.7	22.1
Liver	2011	22 855	67.3	32.7	22.2	16.5	16.3	18.7	17.2	9.2
	2012	26 288	66.3	33.7	22.1	15.7	16.7	18.0	17.4	10.2
	2013	25 814	66.1	33.9	21.3	14.6	17.6	18.7	17.3	10.5
	2014	26 518	66.3	33.7	21.5	13.7	18.1	19.8	16.6	10.3
	2015	26 378	65.7	34.3	20.8	12.8	18.9	19.4	16.5	11.5
	2016	27 212	66.4	33.6	20.3	11.5	20.5	18.6	17.0	12.1
	2017	27 397	65.8	34.2	20.1	11.0	20.2	18.8	17.2	12.7
	2018	26 531	66.5	33.5	19.6	10.3	18.8	19.6	17.8	13.8
	2019	26 582	66.3	33.7	19.4	10.1	16.5	21.1	18.6	14.2
	2020	26 614	66.0	34.0	20.6	9.5	15.1	21.7	18.7	14.4
Gall bladder	2011	103 183	54.5	45.4	34.3	14.0	12.2	13.8	12.8	13.0
	2012	122 513	55.2	44.8	32.9	13.8	12.4	13.9	13.2	13.8
	2013	129 162	55.3	44.7	32.6	12.9	13.0	14.2	13.2	14.0
	2014	131 182	55.6	44.4	32.1	11.8	13.9	14.5	13.2	14.5
	2015	133 126	55.6	44.4	32.0	11.2	15.0	14.1	13.0	14.8
	2016	137 360	55.4	44.6	32.6	10.6	15.5	13.1	12.9	15.3
	2017	138 267	55.6	44.4	32.2	10.2	15.1	13.5	13.2	15.8
	2018	139 844	55.3	44.7	31.8	9.7	14.2	14.2	13.4	16.7
	2019	140 214	55.4	44.6	31.6	9.6	13.3	14.7	13.9	16.9
	2020	134 332	55.9	44.1	31.3	9.1	12.2	15.6	14.0	17.8
Pancreas	2011	13 477	59.9	40.1	20.0	15.6	16.9	19.7	17.7	10.2
	2012	15 550	60.0	40.0	19.8	15.2	17.0	19.5	18.2	10.3
	2013	16 380	59.7	40.3	19.1	13.6	18.0	20.7	17.7	10.9
	2014	17 313	59.5	40.5	18.4	12.4	19.0	21.0	18.2	11.1
	2015	17 407	59.1	40.9	18.2	11.3	19.4	21.6	18.1	11.4
	2016	18 238	58.9	41.1	18.2	10.4	19.9	20.4	19.0	12.2
	2017	19 138	59.2	40.8	17.7	9.9	19.5	19.9	20.1	12.9
	2018	19 152	58.6	41.4	16.9	9.2	18.2	21.5	20.4	13.7
	2019	19 703	58.3	41.7	17.0	9.2	16.5	21.6	21.1	14.6
	2020	19 947	58.1	41.9	16.7	8.4	14.6	22.8	21.9	15.6
Spleen	2011	3609	61.3	38.7	35.3	15.6	14.7	14.8	11.9	7.8
	2012	4142	61.4	38.6	32.9	16.3	15.0	15.1	12.9	7.8
	2013	4509	61.8	38.2	30.8	14.9	15.9	16.5	13.1	8.7
	2014	4272	61.8	38.2	29.9	13.0	17.3	17.0	13.8	9.1
	2015	3568	60.4	39.6	29.7	11.4	17.3	16.6	14.1	10.8
	2016	3171	57.3	42.7	31.9	11.7	17.7	15.7	12.5	10.5
	2017	2864	58.7	41.3	31.6	11.0	18.1	16.0	13.3	10.0
	2018	2544	56.6	43.4	32.6	9.9	15.6	16.9	13.9	11.1
	2019	2413	55.2	44.8	31.3	10.5	16.8	15.8	13.1	12.5
	2020	2096	54.4	45.6	32.8	11.4	12.6	16.7	14.1	12.4
Others	2011	23 218	55.0	45.0	32.0	11.9	11.3	13.3	13.8	17.6
	2012	28 779	55.4	44.6	31.1	11.7	11.7	13.8	13.7	18.0
	2013	36 363	53.1	46.9	28.3	10.9	12.7	14.1	14.8	19.1
	2014	39 854	53.7	46.3	28.1	10.1	13.1	14.5	14.4	19.8
	2015	41 465	53.2	46.8	27.4	9.4	14.0	14.5	14.2	20.6
	2016	43 523	54.0	46.0	27.5	9.2	14.6	13.5	14.0	21.2
	2017	45 622	54.1	45.9	27.0	8.2	14.7	13.5	14.6	21.9
	2018	46 587	54.1	45.9	26.8	8.2	14.0	14.4	14.7	21.9
	2019	50 525	54.8	45.2	27.0	8.1	12.7	15.3	15.0	21.9
	2020	50 048	54.5	45.5	27.2	7.9	11.9	16.0	14.9	22.1
Total	2011	432 532	59.2	40.8	30.6	13.3	12.6	14.4	13.7	15.4
	2012	516 791	59.2	40.8	29.9	13.0	12.8	14.4	13.9	16.0
	2013	545 110	58.8	41.2	29.3	12.1	13.5	14.8	14.0	16.4
	2014	561 391	58.9	41.1	28.7	11.1	14.1	15.2	13.9	17.0
	2015	575 309	58.8	41.2	28.5	10.4	15.0	15.0	13.7	17.4
	2016	584 844	58.7	41.3	28.5	9.8	15.8	14.2	13.7	18.0
	2017	602 437	58.3	41.7	28.1	9.2	15.3	14.3	14.2	18.9
	2018	601 754	58.2	41.8	27.7	8.8	14.4	15.0	14.4	19.6
	2019	609 589	57.9	42.1	27.7	8.6	13.3	15.7	14.9	19.8
	2020	593 088	57.8	42.2	28.0	8.3	12.2	16.5	14.8	20.3

**TABLE 4:** Annual changes in surgeries of each organ by institution type and specialist participation rate

Organ	Year	No. of surgeries	Institution type (%)			Anesthesiologist participation (%)	Board‐certified surgeon participation (%)	Operating surgeon (%)	
			Certified institution	Affiliated institution	Others			Board‐certified surgeon	Non‐board‐ certified surgeon
Esophagus	2011	7246	93.5	5.9	0.6	97.0	87.0	62.8	37.2
	2012	8819	78.1	5.9	16.0	97.4	87.0	62.7	37.3
	2013	8642	90.6	7.1	2.4	97.3	88.4	64.4	35.6
	2014	9021	91.1	6.1	2.8	97.9	90.1	67.6	32.4
	2015	8943	91.5	6.0	2.5	97.9	91.1	69.4	30.6
	2016	9212	92.4	5.0	2.6	98.2	91.2	70.0	30.0
	2017	9359	92.7	4.0	3.3	97.9	92.5	71.8	28.2
	2018	9286	93.8	4.0	2.2	98.5	94.7	75.2	24.8
	2019	9224	94.3	3.8	1.9	98.4	94.2	76.4	23.6
	2020	8713	95.2	3.2	1.5	98.9	95.7	78.3	21.7
Stomach and duodenum	2011	66 740	80.2	17.3	2.6	92.8	69.3	35.1	64.9
	2012	76 186	63.5	15.6	20.9	93.5	70.3	35.6	64.4
	2013	75 583	76.3	19.3	4.4	93.3	73.5	37.7	62.3
	2014	74 920	77.0	18.2	4.8	93.6	75.9	39.2	60.8
	2015	73 877	77.1	18.3	4.6	93.9	76.1	39.2	60.8
	2016	72 234	79.6	16.1	4.3	94.6	78.7	41.0	59.0
	2017	68 287	79.6	15.3	5.1	94.8	79.7	41.8	58.2
	2018	65 152	80.0	14.8	5.1	95.1	81.4	43.2	56.8
	2019	63 610	81.3	14.2	4.5	95.4	83.8	46.1	53.9
	2020	57 171	80.8	14.8	4.4	95.7	85.4	47.6	52.4
Small intestine and colon	2011	151 143	76.8	20.2	2.9	88.1	59.2	25.1	74.9
	2012	184 810	60.6	18.2	21.2	88.9	59.9	25.4	74.6
	2013	198 677	72.6	22.2	5.2	89.6	62.7	26.6	73.4
	2014	206 857	73.0	21.4	5.6	90.8	65.4	28.1	71.9
	2015	214 453	73.8	20.7	5.5	91.6	66.3	28.5	71.5
	2016	218 228	75.6	19.0	5.5	92.4	68.1	29.5	70.5
	2017	235 359	76.0	18.0	6.0	92.9	70.1	31.1	68.9
	2018	236 496	76.3	17.5	6.1	93.3	71.8	32.6	67.4
	2019	239 612	77.1	17.1	5.8	94.1	74.0	33.2	66.8
	2020	238 631	76.5	17.9	5.6	94.5	75.9	34.2	65.8
Rectum and anus	2011	41 061	76.9	19.0	4.1	86.3	68.3	36.9	63.1
	2012	49 704	60.4	18.2	21.4	85.7	68.6	37.6	62.4
	2013	49 980	72.9	21.7	5.4	87.3	71.2	39.4	60.6
	2014	51 454	73.5	20.9	5.6	87.9	73.7	41.6	58.4
	2015	56 092	72.5	20.8	6.7	84.9	73.5	41.5	58.5
	2016	55 666	74.1	19.4	6.6	85.7	74.7	42.1	57.9
	2017	56 144	73.8	18.2	8.0	84.8	76.1	43.9	56.1
	2018	56 162	74.1	17.9	8.0	85.2	77.2	46.7	53.3
	2019	57 706	74.9	17.3	7.8	86.0	80.1	48.9	51.1
	2020	55 536	74.5	18.6	6.8	86.7	81.7	51.0	49.0
Liver	2011	22 855	89.3	9.7	1.1	95.6	85.2	55.2	44.8
	2012	26 288	74.2	9.2	16.7	95.4	85.7	57.4	42.6
	2013	25 814	86.3	10.7	2.9	96.3	87.5	57.1	42.9
	2014	26 518	86.3	10.0	3.7	96.4	89.0	59.6	40.4
	2015	26 378	87.3	9.5	3.2	96.6	89.1	59.1	40.9
	2016	27 212	88.4	8.8	2.9	96.8	90.0	59.6	40.4
	2017	27 397	89.0	7.8	3.1	97.1	91.8	62.5	37.5
	2018	26 531	89.4	7.1	3.5	97.3	92.8	64.1	35.9
	2019	26 582	89.7	6.8	3.6	97.3	94.1	66.4	33.6
	2020	26 614	89.6	7.2	3.1	97.4	94.7	67.7	32.3
Gall bladder	2011	103 183	73.9	22.5	3.6	91.8	61.9	26.4	73.6
	2012	122 513	57.5	19.6	22.9	92.1	62.8	26.3	73.7
	2013	129 162	69.9	24.1	5.9	92.2	65.4	27.3	72.7
	2014	131 182	70.3	23.3	6.4	92.3	67.4	28.1	71.9
	2015	133 126	70.8	22.8	6.4	92.9	68.4	28.1	71.9
	2016	137 360	72.4	21.3	6.3	93.5	69.4	28.9	71.1
	2017	138 267	72.6	20.1	7.3	93.7	71.4	29.9	70.1
	2018	139 844	72.5	20.1	7.4	94.1	73.1	31.1	68.9
	2019	140 214	73.5	19.4	7.1	94.4	75.7	32.3	67.7
	2020	134 332	72.9	20.2	6.9	94.8	77.7	33.8	66.2
Pancreas	2011	13 477	88.1	10.8	1.2	95.8	85.2	57.7	42.3
	2012	15 550	72.8	8.7	18.5	96.3	86.5	59.9	40.1
	2013	16 380	86.5	11.0	2.4	95.9	87.6	60.2	39.8
	2014	17 313	86.9	9.9	3.3	96.2	89.1	61.3	38.7
	2015	17 407	88.4	9.1	2.4	96.4	90.3	61.6	38.4
	2016	18 238	89.8	8.0	2.3	96.8	91.1	62.4	37.6
	2017	19 138	90.4	7.1	2.5	97.2	92.3	63.9	36.1
	2018	19 152	91.3	6.4	2.3	97.3	93.4	66.5	33.5
	2019	19 703	91.9	6.2	1.9	97.2	95.1	69.2	30.8
	2020	19 947	91.9	6.3	1.8	97.6	95.7	70.4	29.6
Spleen	2011	3609	87.0	11.6	1.4	94.6	75.2	44.9	55.1
	2012	4142	70.5	9.5	20.0	81.7	75.8	44.4	55.6
	2013	4509	83.2	13.8	3.0	95.2	75.4	43.3	56.7
	2014	4272	85.4	11.5	3.1	94.6	77.5	45.2	54.8
	2015	3568	85.6	12.3	2.1	94.8	78.9	45.5	54.5
	2016	3171	86.8	10.1	3.1	95.7	80.5	48.0	52.0
	2017	2864	87.4	9.3	3.3	95.3	82.3	49.1	50.9
	2018	2544	86.9	9.7	3.4	95.3	84.7	49.3	50.7
	2019	2413	88.1	8.7	3.2	96.2	86.8	54.0	46.0
	2020	2096	88.6	9.2	2.2	96.5	88.3	55.7	44.3
Others	2011	23 218	80.2	17.0	2.8	90.3	60.4	27.2	72.8
	2012	28 779	65.7	15.2	19.1	91.0	61.1	27.6	72.4
	2013	36 363	76.1	19.3	4.6	91.5	63.4	28.5	71.5
	2014	39 854	76.6	18.2	5.1	91.9	64.9	29.7	70.3
	2015	41 465	78.0	17.2	4.8	92.4	65.6	29.4	70.6
	2016	43 523	79.4	15.8	4.8	92.7	67.3	30.3	69.7
	2017	45 622	80.1	14.8	5.1	93.1	69.7	32.3	67.7
	2018	46 587	80.2	14.2	5.7	93.8	71.2	33.1	66.9
	2019	50 525	80.9	13.9	5.3	94.3	74.0	35.2	64.8
	2020	50 048	80.4	14.7	4.9	94.3	76.1	37.0	63.0

The rates of postoperative complications, 30‐day postoperative mortality, and operative mortality in each organ are shown in Table 5. The incidence of severe postoperative complications in the last decade was less than 5% in the gallbladder and almost 10% in the stomach and duodenum, the small intestine and colon, the rectum and anus, and the liver. Although the rate of severe complications of the esophagus and pancreas increased (4.6% and 7.9% increase in 10 years, respectively), the operative mortality rates for surgery on these organs decreased slightly in this period (Figure 1).

**TABLE 5:** Annual changes in surgeries of each organ by complication and mortality rates

Organ	Year	No. of surgeries	No. of postoperative complications^a^ (%)	No. of postoperative 30‐day mortalities (%)	No. of operative mortalities^b^ (%)
Esophagus	2011	7246	1294 (17.9)	87 (1.2)	279 (3.9)
	2012	8819	1653 (18.7)	117 (1.3)	315 (3.6)
	2013	8642	1593 (18.4)	121 (1.4)	327 (3.8)
	2014	9021	1679 (18.6)	115 (1.3)	289 (3.2)
	2015	8943	1709 (19.1)	103 (1.2)	304 (3.4)
	2016	9212	1805 (19.6)	100 (1.1)	238 (2.6)
	2017	9359	1938 (20.7)	108 (1.2)	208 (2.2)
	2018	9286	2065 (22.2)	108 (1.2)	246 (2.6)
	2019	9224	2035 (22.1)	119 (1.3)	246 (2.7)
	2020	8713	1963 (22.5)	95 (1.1)	212 (2.4)
Stomach and duodenum	2011	66 740	5354 (8.0)	992 (1.5)	2183 (3.3)
	2012	76 186	6447 (8.5)	1085 (1.4)	2381 (3.1)
	2013	75 583	6380 (8.4)	1059 (1.4)	2269 (3.0)
	2014	74 920	6328 (8.4)	1064 (1.4)	2174 (2.9)
	2015	73 877	6418 (8.7)	1007 (1.4)	2110 (2.9)
	2016	72 234	6413 (8.9)	1066 (1.5)	2016 (2.8)
	2017	68 287	6455 (9.5)	1046 (1.5)	1863 (2.7)
	2018	65 152	6228 (9.6)	1048 (1.6)	1833 (2.8)
	2019	63 610	6159 (9.7)	1022 (1.6)	1826 (2.9)
	2020	57 171	5849 (10.2)	977 (1.7)	1679 (2.9)
Small intestine and colon	2011	151 143	12 184 (8.1)	2943 (1.9)	5390 (3.6)
	2012	184 810	15 395 (8.3)	3564 (1.9)	6583 (3.6)
	2013	198 677	16 709 (8.4)	3723 (1.9)	6803 (3.4)
	2014	206 857	17 776 (8.6)	3822 (1.9)	6961 (3.4)
	2015	214 453	18 372 (8.6)	4019 (1.9)	7092 (3.3)
	2016	218 228	19 020 (8.7)	3933 (1.8)	6621 (3.0)
	2017	235 359	21 854 (9.3)	4588 (1.9)	7118 (3.0)
	2018	236 496	21 881 (9.3)	4452 (1.9)	7116 (3.0)
	2019	239 612	22 061 (9.2)	4671 (1.9)	7298 (3.0)
	2020	238 631	22 344 (9.4)	4791 (2.0)	7261 (3.0)
Rectum and anus	2011	41 061	3584 (8.7)	395 (1.0)	676 (1.6)
	2012	49 704	4488 (9.0)	462 (0.9)	802 (1.6)
	2013	49 980	4684 (9.4)	517 (1.0)	858 (1.7)
	2014	51 454	4711 (9.2)	449 (0.9)	792 (1.5)
	2015	56 092	4986 (8.9)	519 (0.9)	824 (1.5)
	2016	55 666	5194 (9.3)	503 (0.9)	766 (1.4)
	2017	56 144	5600 (10.0)	556 (1.0)	829 (1.5)
	2018	56 162	5622 (10.0)	522 (0.9)	803 (1.4)
	2019	57 706	5573 (9.7)	563 (1.0)	839 (1.5)
	2020	55 536	5383 (9.7)	555 (1.0)	797 (1.4)
Liver	2011	22 855	1933 (8.5)	309 (1.4)	590 (2.6)
	2012	26 288	2454 (9.3)	310 (1.2)	605 (2.3)
	2013	25 814	2549 (9.9)	275 (1.1)	575 (2.2)
	2014	26 518	2466 (9.3)	246 (0.9)	481 (1.8)
	2015	26 378	2537 (9.6)	234 (0.9)	451 (1.7)
	2016	27 212	2543 (9.3)	222 (0.8)	382 (1.4)
	2017	27 397	2724 (9.9)	214 (0.8)	364 (1.3)
	2018	26 531	2737 (10.3)	189 (0.7)	372 (1.4)
	2019	26 582	2624 (9.9)	201 (0.8)	334 (1.3)
	2020	26 614	2804 (10.5)	338 (1.3)	475 (1.8)
Gall bladder	2011	103 183	3473 (3.4)	483 (0.5)	946 (0.9)
	2012	122 513	4587 (3.7)	531 (0.4)	1082 (0.9)
	2013	129 162	4982 (3.9)	546 (0.4)	1130 (0.9)
	2014	131 182	5020 (3.8)	569 (0.4)	1097 (0.8)
	2015	133 126	5231 (3.9)	541 (0.4)	1036 (0.8)
	2016	137 360	5320 (3.9)	559 (0.4)	980 (0.7)
	2017	138 267	5761 (4.2)	576 (0.4)	968 (0.7)
	2018	139 844	5964 (4.3)	584 (0.4)	954 (0.7)
	2019	140 214	5748 (4.1)	565 (0.4)	935 (0.7)
	2020	134 332	5888 (4.4)	620 (0.5)	978 (0.7)
Pancreas	2011	13 477	1994 (14.8)	175 (1.3)	386 (2.9)
	2012	15 550	2595 (16.7)	213 (1.4)	437 (2.8)
	2013	16 380	2917 (17.8)	211 (1.3)	482 (2.9)
	2014	17 313	2966 (17.1)	195 (1.1)	423 (2.4)
	2015	17 407	3229 (18.6)	185 (1.1)	379 (2.2)
	2016	18 238	3543 (19.4)	185 (1.0)	390 (2.1)
	2017	19 138	4076 (21.3)	219 (1.1)	365 (1.9)
	2018	19 152	4309 (22.5)	178 (0.9)	325 (1.7)
	2019	19 703	4522 (23.0)	199 (1.0)	335 (1.7)
	2020	19 947	4520 (22.7)	205 (1.0)	345 (1.7)
Spleen	2011	3609	400 (11.1)	83 (2.3)	137 (3.8)
	2012	4142	528 (12.7)	84 (2.0)	138 (3.3)
	2013	4509	575 (12.8)	79 (1.8)	139 (3.1)
	2014	4272	549 (12.9)	88 (2.1)	137 (3.2)
	2015	3568	543 (15.2)	88 (2.5)	144 (4.0)
	2016	3171	449 (14.2)	76 (2.4)	117 (3.7)
	2017	2864	434 (15.2)	65 (2.3)	89 (3.1)
	2018	2544	418 (16.4)	69 (2.7)	104 (4.1)
	2019	2413	380 (15.7)	71 (2.9)	97 (4.0)
	2020	2096	313 (14.9)	63 (3.0)	87 (4.2)
Others	2011	23 218	3494 (15.0)	1163 (5.0)	1887 (8.1)
	2012	28 779	4388 (15.2)	1399 (4.9)	2293 (8.0)
	2013	36 363	4712 (13.0)	1401 (3.9)	2346 (6.5)
	2014	39 854	5176 (13.0)	1521 (3.8)	2489 (6.2)
	2015	41 465	5380 (13.0)	1541 (3.7)	2545 (6.1)
	2016	43 523	5975 (13.7)	1760 (4.0)	2684 (6.2)
	2017	45 622	6539 (14.3)	1909 (4.2)	2699 (5.9)
	2018	46 587	6645 (14.3)	1865 (4.0)	2710 (5.8)
	2019	50 525	7750 (15.3)	2221 (4.4)	3220 (6.4)
	2020	50 048	7838 (15.7)	2267 (4.5)	3284 (6.6)

^a^ Complications were defined by Clavien–Dindo grade IIIa–V.

^b^ Operative mortality was a rate that combined 30‐day mortality and hospitalization death in 31–90 days after surgery.

### 3.3 Annual changes in surgeries of the eight major operative procedures

**FIGURE 2:**
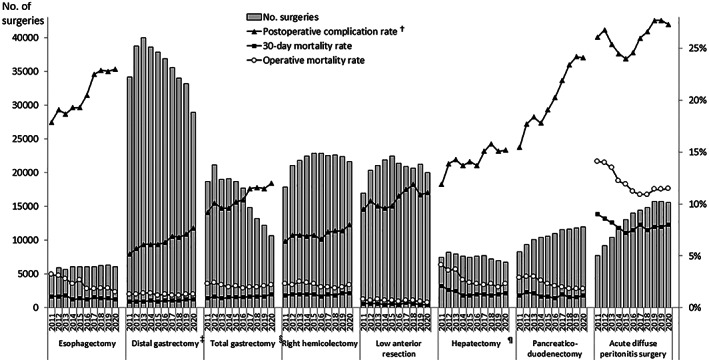
Annual changes in number of the surgeries, 30‐day and operative mortality rates, and complication rates of the eight major surgical procedures. ^†^Postoperative complication was defined as grades IIIa–V in the Clavien–Dindo classification. ^‡^Including *pylorus‐preserving gastrectomy* and *segmental gastrectomy*, ^§^including *proximal gastrectomy*, ^¶^segmentectomy or more; excluding *lateral segmentectomy*

As shown in Figure 2, the number of surgeries of the eight major operative procedures in 2020 decreased from 2019 (esophagectomy, 3.0% decrease year‐on‐year; distal gastrectomy, 12.8%; total gastrectomy, 12.6%; right hemicolectomy, 3.5%; low anterior resection, 5.8%; hepatectomy, 3.8%; acute diffuse peritonitis surgery, 1.4%), except for pancreaticoduodenectomy (1.2% increase year‐on‐year).

The ratio of age ≥80 years increased 2.0%–7.0% in the last decade (Table 6). In terms of the institution types, most cases (94%–99% in 2020) of each procedure were performed in certified or affiliated institutions of the JSGS (Table 7). In 2020, the percentages of surgeries with participation of an anesthesiologist in each procedure were generally more than 95%. Board‐certified surgeon participation rates have been increasing year by year for all procedures. The percentage of the participation of JSGS board‐certified surgeons in 2020 was 96% to 98% for esophagectomy, hepatectomy, and pancreaticoduodenectomy. In contrast, the percentages were relatively low for acute diffuse peritonitis surgery (76.1% in 2020) (Table 7).

**TABLE 6:** Annual changes in surgeries of the eight major operative procedures by sex and age of patients

Procedure	Year	No. of surgeries	Sex (%)		Age (%)					
			Male	Female	<60	60 to <65	65 to <70	70 to <75	75 to <80	≥80
Esophagectomy	2011	4916	84.1	15.9	20.4	20.8	22.5	19.4	12.2	4.7
	2012	5946	84.4	15.6	19.7	21.3	20.7	20.3	13.1	4.9
	2013	5694	83.6	16.4	18.3	18.3	22.6	21.3	13.8	5.8
	2014	6091	84.0	16.0	18.7	17.8	22.8	22.0	13.4	5.2
	2015	6060	82.9	17.1	17.9	16.3	23.6	23.5	13.1	5.7
	2016	6041	81.7	18.3	17.8	15.8	25.3	21.6	14.3	5.2
	2017	6100	82.3	17.7	17.0	14.6	25.6	20.6	15.8	6.3
	2018	6207	80.5	19.5	17.2	14.2	22.6	22.8	16.8	6.5
	2019	6298	81.0	19.0	17.0	13.9	20.7	24.1	17.2	7.0
	2020	6111	80.4	19.6	17.0	14.5	19.5	24.0	16.8	8.2
Distal gastrectomy^a^	2011	34 160	66.6	33.4	18.1	15.0	14.2	17.4	16.8	18.5
	2012	38 750	66.9	33.1	16.9	14.8	15.0	17.8	16.5	18.8
	2013	39 957	66.7	33.3	16.3	13.5	15.8	17.8	17.6	19.0
	2014	38 584	66.4	33.6	15.7	12.4	16.6	18.4	17.3	19.5
	2015	37 819	66.6	33.4	14.8	11.3	17.5	18.2	17.5	20.6
	2016	36 852	66.6	33.4	14.5	10.4	18.5	17.6	17.4	21.6
	2017	35 517	66.8	33.2	13.4	9.9	18.0	18.1	18.0	22.6
	2018	33 988	66.6	33.4	12.9	9.1	16.9	19.0	18.1	24.0
	2019	33 177	66.5	33.5	12.2	8.6	15.3	20.4	19.3	24.3
	2020	28 944	67.1	32.9	11.6	8.1	14.1	21.2	19.6	25.4
Total gastrectomy^b^	2011	18 652	73.7	26.3	16.6	14.7	16.0	19.7	18.0	15.0
	2012	21 122	74.2	25.8	15.5	14.8	15.7	19.2	18.5	16.3
	2013	19 035	74.0	26.0	14.7	13.5	16.9	19.4	19.2	16.3
	2014	19 071	73.7	26.3	14.0	12.3	17.2	20.1	18.9	17.5
	2015	18 695	74.5	25.5	13.7	11.1	18.9	20.8	18.2	17.4
	2016	17 670	74.4	25.6	12.6	10.3	19.6	19.5	19.0	19.0
	2017	14 840	74.2	25.8	12.2	9.9	19.0	19.6	19.8	19.5
	2018	13 223	74.4	25.6	10.8	9.1	18.0	20.6	20.6	20.9
	2019	12 188	74.3	25.7	10.7	9.0	16.9	21.4	21.5	20.6
	2020	10 652	75.2	24.8	10.6	8.0	15.3	23.5	20.6	22.0
Right hemicolectomy	2011	17 890	50.5	49.5	12.8	11.6	13.1	17.3	18.8	26.5
	2012	21 034	50.3	49.7	13.1	10.9	13.1	17.0	19.0	26.9
	2013	21 814	50.6	49.4	13.0	10.0	13.4	17.6	18.9	27.1
	2014	22 446	50.6	49.4	12.0	9.2	13.8	18.2	18.6	28.2
	2015	22 850	50.5	49.5	11.5	8.6	14.6	18.1	18.1	29.1
	2016	22 829	51.3	48.7	11.4	7.7	15.9	16.7	18.5	29.8
	2017	22 543	50.9	49.1	11.3	7.4	14.9	16.3	19.3	30.8
	2018	22 610	51.4	48.6	10.7	6.9	13.9	17.7	19.6	31.2
	2019	22 410	51.5	48.5	11.0	6.6	12.9	17.7	19.7	32.1
	2020	21 626	51.1	48.9	11.4	6.4	11.2	18.3	19.8	32.9
Low anterior resection	2011	16 984	64.8	35.2	24.1	18.5	16.5	16.2	12.9	11.7
	2012	20 321	64.8	35.2	24.2	17.6	16.5	16.6	13.1	12.0
	2013	21 096	64.2	35.8	23.8	16.5	17.4	16.9	13.5	11.8
	2014	21 861	64.8	35.2	23.1	15.7	18.3	17.9	13.1	11.9
	2015	22 493	64.4	35.6	23.5	14.2	19.6	17.1	13.6	12.0
	2016	21 387	64.4	35.6	23.4	13.6	20.7	16.8	13.2	12.2
	2017	20 879	64.2	35.8	23.2	12.6	20.9	16.7	13.5	13.2
	2018	20 636	64.9	35.1	22.9	12.5	19.3	18.0	14.4	12.9
	2019	21 262	63.9	36.1	23.3	11.6	18.4	18.6	14.6	13.5
	2020	20 022	63.9	36.1	23.9	11.9	16.6	19.8	14.2	13.7
Hepatectomy^c^	2011	7434	70.4	29.6	20.1	16.4	16.5	20.4	18.0	8.7
	2012	8239	69.5	30.5	19.8	16.1	17.4	19.5	18.5	8.8
	2013	7937	69.4	30.6	19.4	14.2	18.0	20.3	18.2	9.9
	2014	7666	69.2	30.8	18.5	13.8	18.5	21.5	17.6	10.0
	2015	7439	68.9	31.1	18.7	12.5	19.3	20.9	17.6	11.1
	2016	7610	68.7	31.3	18.0	11.9	21.1	20.4	17.5	11.1
	2017	7698	69.5	30.5	17.2	11.3	20.5	20.4	18.7	11.9
	2018	7192	69.5	30.5	17.2	9.6	19.1	21.4	19.4	13.3
	2019	7018	69.2	30.8	16.7	9.2	16.8	22.6	20.9	13.8
	2020	6753	68.4	31.6	16.7	9.3	15.4	22.8	21.1	14.7
Pancreaticoduodenectomy	2011	8305	61.9	38.1	16.1	16.0	17.3	20.9	18.8	10.9
	2012	9329	62.0	38.0	14.7	15.8	18.0	20.6	20.2	10.6
	2013	10 068	60.9	39.1	14.0	12.6	19.6	22.5	19.4	11.8
	2014	10 400	59.5	40.5	18.4	12.4	19.0	21.0	18.2	11.1
	2015	10 576	60.7	39.3	14.2	11.7	20.0	22.9	19.3	12.0
	2016	11 028	61.1	38.9	14.2	10.3	20.6	21.8	20.3	12.7
	2017	11 580	61.1	38.9	13.8	9.8	20.4	20.8	21.6	13.6
	2018	11 626	60.3	39.7	13.3	9.1	18.9	22.2	22.0	14.6
	2019	11 813	60.7	39.3	13.1	9.1	17.4	22.6	22.1	15.6
	2020	11 953	60.6	39.4	12.9	8.5	15.4	24.1	23.1	16.0
Acute diffuse peritonitis surgery	2011	7753	60.0	40.0	31.4	11.2	9.7	11.7	13.2	22.9
	2012	9177	61.0	39.0	30.3	11.2	10.1	11.6	13.4	23.4
	2013	10 447	60.1	39.9	29.1	10.3	11.5	11.8	13.1	24.1
	2014	12 085	61.2	38.8	28.4	9.5	12.2	12.3	12.9	24.7
	2015	13 030	59.4	40.6	28.2	8.9	12.5	13.1	12.3	25.0
	2016	13 981	60.2	39.8	27.4	8.6	13.4	12.4	12.3	26.0
	2017	14 423	59.4	40.6	26.5	7.8	13.0	12.0	13.6	27.1
	2018	14 835	59.2	40.8	26.1	7.7	12.7	13.1	13.5	26.9
	2019	15 765	59.2	40.8	25.2	7.7	11.6	13.6	14.1	27.7
	2020	15 542	58.9	41.1	25.4	7.6	11.0	14.2	14.0	27.8

^a^ Including *pylorus preserving gastrectomy* and *segmental gastrectomy*.

^b^ Including *proximal gastrectomy*.

^c^ Segmentectomy or more; excluding *lateral segmentectomy*.

**TABLE 7:** Annual changes in surgeries of the eight major operative procedures by institution type and specialist participation rate

Procedure	Year	No. of surgeries	Institution type (%)			Anesthesiologist participation (%)	Board‐certified surgeon participation (%)	Operating surgeon (%)	
			Certified institution	Affiliated institution	Others			Board‐ certified surgeon	Non‐board‐ certified surgeon
Esophagectomy	2011	4916	94.2	5.3	0.5	97.6	88.4	63.5	36.5
	2012	5946	78.3	4.9	16.8	98.1	89.0	64.8	35.2
	2013	5694	92.9	5.9	1.2	98.0	90.8	66.6	33.4
	2014	6091	93.6	4.7	1.7	98.6	92.6	70.2	29.8
	2015	6060	93.6	4.6	1.8	98.5	93.5	72.1	27.9
	2016	6041	94.5	3.8	1.7	98.8	93.7	73.2	26.8
	2017	6100	95.3	3.1	1.7	98.8	94.8	74.7	25.3
	2018	6207	95.9	2.7	1.4	99.1	96.6	78.8	21.2
	2019	6298	96.3	2.3	1.5	98.9	96.4	80.6	19.4
	2020	6111	96.9	2.1	1.0	99.3	98.0	82.1	17.9
Distal gastrectomy^a^	2011	34 160	81.1	16.6	2.3	93.2	71.3	37.0	63.0
	2012	38 750	64.5	15.2	20.3	93.9	72.5	37.9	62.1
	2013	39 957	76.6	19.2	4.1	93.6	76.1	40.6	59.4
	2014	38 584	77.7	17.8	4.5	94.0	78.4	42.1	57.9
	2015	37 819	77.3	18.3	4.4	94.1	78.1	41.3	58.7
	2016	36 852	80.2	15.9	4.0	95.0	81.8	43.8	56.2
	2017	35 517	80.2	14.9	4.8	95.4	82.4	45.2	54.8
	2018	33 988	80.7	14.4	4.8	95.6	84.2	46.6	53.4
	2019	33 177	82.4	13.5	4.0	95.7	86.4	50.1	49.9
	2020	28 944	81.6	14.5	3.9	96.1	87.9	51.4	48.6
Total gastrectomy^b^	2011	18 652	80.9	16.8	2.3	93.9	71.6	37.4	62.6
	2012	21 122	63.0	15.3	21.7	94.3	72.1	38.0	62.0
	2013	19 035	77.2	18.9	3.9	94.2	75.0	39.5	60.5
	2014	19 071	77.8	17.9	4.3	94.4	77.7	41.7	58.3
	2015	18 695	77.9	17.9	4.1	94.5	78.2	42.6	57.4
	2016	17 670	80.0	15.9	4.0	95.0	81.4	45.0	55.0
	2017	14 840	79.3	15.8	4.9	95.0	80.7	44.3	55.7
	2018	13 223	79.6	15.5	4.9	95.4	82.6	46.2	53.8
	2019	12 188	80.0	15.5	4.4	95.7	85.5	49.2	50.8
	2020	10 652	79.0	16.4	4.6	96.0	86.7	51.8	48.2
Right hemicolectomy	2011	17 890	75.7	21.2	3.1	92.7	66.0	30.5	69.5
	2012	21 034	60.0	18.3	21.7	93.0	67.1	30.8	69.2
	2013	21 814	72.1	22.3	5.6	92.9	69.7	32.6	67.4
	2014	22 446	71.2	23.1	5.7	93.4	71.9	33.6	66.4
	2015	22 850	72.1	22.0	5.9	94.1	72.4	33.5	66.5
	2016	22 829	73.8	20.1	6.1	94.5	74.2	34.3	65.7
	2017	22 543	75.0	18.4	6.6	94.7	76.4	37.1	62.9
	2018	22 610	74.8	19.0	6.2	94.7	77.8	38.2	61.8
	2019	22 410	75.8	18.1	6.1	95.6	80.1	39.2	60.8
	2020	21 626	74.6	19.3	6.1	95.4	81.5	40.3	59.7
Low anterior resection	2011	16 984	79.4	17.7	2.9	93.4	72.7	41.6	58.4
	2012	20 321	64.0	16.2	19.7	93.8	73.0	42.3	57.7
	2013	21 096	76.3	19.5	4.2	93.7	75.5	44.3	55.7
	2014	21 861	76.2	19.0	4.9	94.4	78.2	47.2	52.8
	2015	22 493	76.9	18.3	4.8	94.6	79.2	47.7	52.3
	2016	21 387	79.0	16.4	4.7	95.0	81.0	48.8	51.2
	2017	20 879	79.3	15.6	5.1	95.2	83.1	51.2	48.8
	2018	20 636	80.9	14.3	4.8	95.2	84.5	54.4	45.6
	2019	21 262	81.2	14.1	4.6	95.6	86.8	58.3	41.7
	2020	20 022	80.3	15.4	4.3	96.1	88.2	60.6	39.4
Hepatectomy^c^	2011	7434	91.1	8.0	0.8	96.4	88.9	61.5	38.5
	2012	8239	75.9	7.9	16.3	96.8	89.3	64.0	36.0
	2013	7937	88.1	9.7	2.2	96.9	91.0	65.2	34.8
	2014	7666	88.2	8.7	3.1	96.7	92.3	66.6	33.4
	2015	7439	89.2	8.6	2.2	97.2	92.3	66.6	33.4
	2016	7610	90.7	7.1	2.1	97.1	93.3	67.7	32.3
	2017	7698	91.2	6.6	2.2	97.7	95.1	72.3	27.7
	2018	7192	92.8	5.2	2.0	97.7	95.8	72.8	27.2
	2019	7018	92.7	5.2	2.1	97.8	96.3	74.2	25.8
	2020	6753	91.8	6.1	2.0	98.1	97.3	76.5	23.5
Pancreaticoduodenectomy	2011	8305	87.8	11.0	1.2	95.9	85.7	58.7	41.3
	2012	9329	72.4	8.8	18.8	96.6	87.2	60.9	39.1
	2013	10 068	85.9	11.7	2.4	96.0	87.9	60.5	39.5
	2014	10 400	86.4	10.4	3.3	96.4	90.3	62.2	37.8
	2015	10 576	88.5	9.2	2.4	96.9	90.9	62.1	37.9
	2016	11 028	89.4	8.3	2.3	97.1	91.7	63.3	36.7
	2017	11 580	90.5	7.2	2.3	97.3	93.0	65.0	35.0
	2018	11 626	91.4	6.4	2.2	97.4	94.0	67.6	32.4
	2019	11 813	92.0	6.2	1.9	97.2	95.5	69.6	30.4
	2020	11 953	91.8	6.4	1.7	97.9	96.4	71.2	28.8
Acute diffuse peritonitis surgery	2011	7753	80.6	16.9	2.4	90.0	58.5	23.5	76.5
	2012	9177	65.2	16.4	18.4	90.4	59.4	22.7	77.3
	2013	10 447	77.7	18.1	4.2	91.2	62.4	23.9	76.1
	2014	12 085	77.7	17.2	5.1	91.9	63.3	25.1	74.9
	2015	13 030	79.8	15.9	4.3	92.2	64.5	24.9	75.1
	2016	13 981	82.2	13.8	4.0	93.0	66.8	26.1	73.9
	2017	14 423	83.1	13.0	3.8	93.3	69.0	27.2	72.8
	2018	14 835	83.4	12.4	4.2	93.6	70.4	28.7	71.3
	2019	15 765	83.8	12.2	4.0	94.6	73.7	29.8	70.2
	2020	15 542	82.0	13.8	4.2	94.6	76.1	31.7	68.3

^a^ Including *pylorus preserving gastrectomy* and *segmental gastrectomy*.

^b^ Including *proximal gastrectomy*.

^c^ Segmentectomy or more; excluding *lateral segmentectomy*.

The rates of postoperative complications, re‐operation, 30‐day postoperative mortality, and operative mortality in the eight major operative procedures are shown in Table 8. The incidences of severe complications in these procedures, except acute diffuse peritonitis surgery, were on a rising trend in the past decade. Meanwhile, the re‐operation rates of all eight procedures were stable in the same period, and the 30‐day mortality rates of these procedures also remained relatively constant for all procedures. The operative mortality rates of esophagectomy, hepatectomy, pancreaticoduodenectomy, and acute diffuse peritonitis surgery in 2020 decreased by 1.1%–2.6% from 2011.

**TABLE 8:** Annual changes in surgeries of the eight major operative procedures by complication and mortality rates

Procedure	Year	No. of surgeries	No. of postoperative complications^a^ (%)	No. of re‐operations (%)	No. of postoperative 30‐day mortalities (%)	No. of operative mortalities^b^ (%)
Esophagectomy	2011	4916	879 (17.9)	310 (6.3)	55 (1.1)	158 (3.2)
	2012	5946	1135 (19.1)	345 (5.8)	63 (1.1)	183 (3.1)
	2013	5694	1067 (18.7)	375 (6.6)	67 (1.2)	161 (2.8)
	2014	6091	1178 (19.3)	367 (6.0)	49 (0.8)	140 (2.3)
	2015	6060	1171 (19.3)	392 (6.5)	57 (0.9)	166 (2.7)
	2016	6041	1240 (20.5)	357 (5.9)	49 (0.8)	109 (1.8)
	2017	6100	1374 (22.5)	355 (5.8)	61 (1.0)	108 (1.8)
	2018	6207	1420 (22.9)	367 (5.9)	53 (0.9)	115 (1.9)
	2019	6298	1435 (22.8)	353 (5.6)	54 (0.9)	120 (1.9)
	2020	6111	1403 (23.0)	384 (6.3)	47 (0.8)	92 (1.5)
Distal gastrectomy^c^	2011	34 160	1774 (5.2)	709 (2.1)	208 (0.6)	451 (1.3)
	2012	38 750	2205 (5.7)	849 (2.2)	232 (0.6)	516 (1.3)
	2013	39 957	2450 (6.1)	892 (2.2)	239 (0.6)	542 (1.4)
	2014	38 584	2356 (6.1)	941 (2.4)	264 (0.7)	523 (1.4)
	2015	37 819	2325 (6.1)	851 (2.3)	222 (0.6)	452 (1.2)
	2016	36 852	2314 (6.3)	825 (2.2)	249 (0.7)	473 (1.3)
	2017	35 517	2445 (6.9)	859 (2.4)	253 (0.7)	437 (1.2)
	2018	33 988	2327 (6.8)	737 (2.2)	227 (0.7)	393 (1.2)
	2019	33 177	2361 (7.1)	739 (2.2)	253 (0.8)	427 (1.3)
	2020	28 944	2235 (7.7)	721 (2.5)	238 (0.8)	387 (1.3)
Total gastrectomy^d^	2011	18 652	1716 (9.2)	634 (3.4)	177 (0.9)	427 (2.3)
	2012	21 122	2135 (10.1)	758 (3.6)	224 (1.1)	503 (2.4)
	2013	19 035	1831 (9.6)	642 (3.4)	169 (0.9)	428 (2.2)
	2014	19 071	1840 (9.6)	698 (3.7)	185 (1.0)	379 (2.0)
	2015	18 695	1907 (10.2)	654 (3.5)	178 (1.0)	387 (2.1)
	2016	17 670	1835 (10.4)	638 (3.6)	174 (1.0)	336 (1.9)
	2017	14 840	1702 (11.5)	514 (3.5)	161 (1.1)	293 (2.0)
	2018	13 223	1529 (11.6)	487 (3.7)	148 (1.1)	265 (2.0)
	2019	12 188	1406 (11.5)	427 (3.5)	136 (1.1)	258 (2.1)
	2020	10 652	1275 (12.0)	425 (4.0)	137 (1.3)	230 (2.2)
Right hemicolectomy	2011	17 890	1150 (6.4)	588 (3.3)	213 (1.2)	410 (2.3)
	2012	21 034	1470 (7.0)	677 (3.2)	263 (1.3)	471 (2.2)
	2013	21 814	1527 (7.0)	721 (3.3)	280 (1.3)	538 (2.5)
	2014	22 446	1544 (6.9)	771 (3.4)	287 (1.3)	530 (2.4)
	2015	22 850	1607 (7.0)	769 (3.4)	301 (1.3)	534 (2.3)
	2016	22 829	1510 (6.6)	791 (3.5)	253 (1.1)	449 (2.0)
	2017	22 543	1648 (7.3)	785 (3.5)	296 (1.3)	450 (2.0)
	2018	22 610	1679 (7.4)	740 (3.3)	276 (1.2)	424 (1.9)
	2019	22 410	1666 (7.4)	713 (3.2)	306 (1.4)	449 (2.0)
	2020	21 626	1724 (8.0)	713 (3.3)	313 (1.4)	471 (2.2)
Low anterior resection	2011	16 984	1616 (9.5)	1213 (7.1)	75 (0.4)	136 (0.8)
	2012	20 321	2092 (10.3)	1413 (6.9)	88 (0.4)	149 (0.7)
	2013	21 096	2059 (9.8)	1473 (7.0)	80 (0.4)	175 (0.8)
	2014	21 861	2098 (9.6)	1546 (7.1)	70 (0.3)	152 (0.7)
	2015	22 493	2210 (9.8)	1550 (6.9)	95 (0.4)	156 (0.7)
	2016	21 387	2306 (10.8)	1492 (7.0)	68 (0.3)	126 (0.6)
	2017	20 879	2376 (11.4)	1330 (6.4)	96 (0.5)	148 (0.7)
	2018	20 636	2454 (11.9)	1424 (6.9)	90 (0.4)	142 (0.7)
	2019	21 262	2320 (10.9)	1346 (6.3)	73 (0.3)	119 (0.6)
	2020	20 022	2229 (11.1)	1341 (6.7)	69 (0.3)	102 (0.5)
Hepatectomy^e^	2011	7434	886 (11.9)	203 (2.7)	155 (2.1)	303 (4.1)
	2012	8239	1146 (13.9)	248 (3.0)	142 (1.7)	293 (3.6)
	2013	7937	1135 (14.3)	226 (2.8)	130 (1.6)	290 (3.7)
	2014	7666	1052 (13.7)	242 (3.2)	94 (1.2)	208 (2.7)
	2015	7439	1049 (14.1)	213 (2.9)	87 (1.2)	182 (2.4)
	2016	7610	1046 (13.7)	220 (2.9)	96 (1.3)	178 (2.3)
	2017	7698	1160 (15.1)	221 (2.9)	97 (1.3)	169 (2.2)
	2018	7192	1137 (15.8)	211 (2.9)	83 (1.2)	163 (2.3)
	2019	7018	1058 (15.1)	189 (2.7)	94 (1.3)	143 (2.0)
	2020	6753	1027 (15.2)	202 (3.0)	93 (1.4)	155 (2.3)
Pancreaticoduodenectomy	2011	8305	1285 (15.5)	299 (3.6)	97 (1.2)	238 (2.9)
	2012	9329	1654 (17.7)	365 (3.9)	137 (1.5)	281 (3.0)
	2013	10 068	1853 (18.4)	407 (4.0)	142 (1.4)	307 (3.0)
	2014	10 400	1847 (17.8)	374 (3.6)	111 (1.1)	267 (2.6)
	2015	10 576	2025 (19.1)	378 (3.6)	120 (1.1)	247 (2.3)
	2016	11 028	2242 (20.3)	393 (3.6)	98 (0.9)	232 (2.1)
	2017	11 580	2539 (21.9)	413 (3.6)	145 (1.3)	232 (2.0)
	2018	11 626	2716 (23.4)	402 (3.5)	111 (1.0)	204 (1.8)
	2019	11 813	2854 (24.2)	402 (3.4)	119 (1.0)	210 (1.8)
	2020	11 953	2882 (24.1)	404 (3.4)	138 (1.2)	216 (1.8)
Acute diffuse peritonitis surgery	2011	7753	2022 (26.1)	634 (8.2)	697 (9.0)	1096 (14.1)
	2012	9177	2456 (26.8)	685 (7.5)	785 (8.6)	1289 (14.0)
	2013	10 447	2652 (25.4)	786 (7.5)	861 (8.2)	1408 (13.5)
	2014	12 085	2966 (24.5)	937 (7.8)	927 (7.7)	1472 (12.2)
	2015	13 030	3126 (24.0)	1051 (8.1)	943 (7.2)	1551 (11.9)
	2016	13 981	3445 (24.6)	1068 (7.6)	1052 (7.5)	1572 (11.2)
	2017	14 423	3756 (26.0)	1125 (7.8)	1152 (8.0)	1575 (10.9)
	2018	14 835	3943 (26.6)	1183 (8.0)	1117 (7.5)	1617 (10.9)
	2019	15 765	4367 (27.7)	1247 (7.9)	1233 (7.8)	1795 (11.4)
	2020	15 542	4239 (27.3)	1208 (7.8)	1247 (8.0)	1794 (11.5)

^a^ Complications were defined by Clavien–Dindo grade IIIa–V.

^b^ Operative mortality was a rate that combined 30‐day mortality and hospitalization death in 31–90 days after surgery.

^c^ Including *pylorus preserving gastrectomy* and *segmental gastrectomy*.

^d^ Including *proximal gastrectomy*.

^e^ Segmentectomy or more; excluding *lateral segmentectomy*.

**FIGURE 3:**
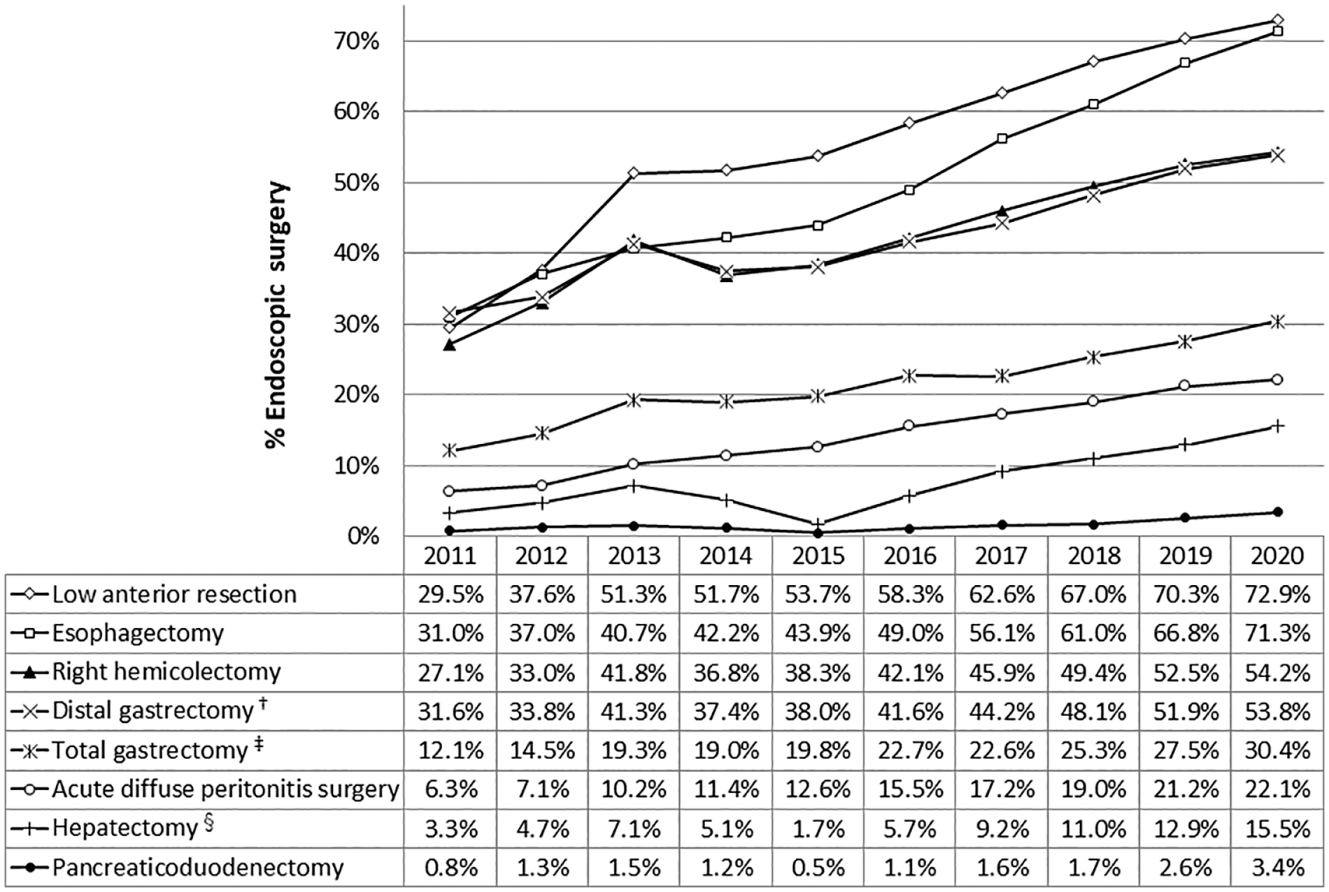
Annual changes in the rate of endoscopic surgery in the eight major surgical procedures. ^†^Including *pylorus‐preserving gastrectomy* and *segmental gastrectomy*, ^‡^including *proximal gastrectomy*, ^§^segmentectomy or more; excluding *lateral segmentectomy*

The rates of endoscopic surgery for the eight major operative procedures are shown in Figure 3. From 2015, endoscopic surgery increased at a constant rate for each in all eight procedures. The endoscopic surgery rate of esophagectomy reached over 70% in 2020 and almost caught up with that of low anterior resection.

## 4 DISCUSSION

This is the first annual report of each operative procedure in the latest version of the “Training Curriculum for Board‐Certified Surgeons in Gastroenterology,” which was partially revised in 2019. The four procedures, i.e., surgery for hepatic trauma, surgery for bile duct trauma, surgery for pancreatic trauma and pancreatic transplantation, were newly registrable in the NCD from 2019. All three, hepatic, biliary, and pancreatic surgeries for trauma, showed high operative mortalities (27.5% for hepatic trauma, 16.7% for pancreatic trauma, and 9.8% for bile duct trauma) in 2020, and the rates of participation of JSGS board‐certified surgeons were in inverse relation to the mortalities (participation rate: 63.4%, 77.8%, and 83.6%, respectively). To improve the outcomes of these surgical procedures, continuous accumulation and analysis of further data regarding short‐term outcomes and patients' characteristics are warranted.

There were seven high mortality procedures that were performed in over 100 cases in 2020, with the exception of trauma surgeries. Of these, five procedures (gastrointestinal perforation closure, total colectomy, external cholecystostomy, esophageal fistula construction, and acute diffuse peritonitis surgery) constantly showed high mortalities, at a rate of more than 10%, in recent years.^24^, ^25^ The difficulty levels of these procedures are defined as moderate or low in the JSGS, and the rate of participation of board‐certified surgeons was relatively low (approximately 75%, except for esophageal fistula construction). Most of them were emergency surgeries and were considered to have included many patients in a moribund condition.

In this report, the 10‐year data of the gastroenterological section of the NCD were also summarized. The total number of gastroenterological surgeries registered in the NCD has gradually increased in the last 10 years, except for the year 2020. The first patient with novel coronavirus disease 2019 (COVID‐19) in Japan was diagnosed in January 2020, and then COVID‐19 infection spread rapidly in this country as a pandemic.^29^ The annual total number of gastroenterological surgeries was decreased by 2.7% in 2020 compared to the prior year. However, this reduction rate was relatively less compared to those reported for surgeries in some other fields (e.g., coronary artery bypass grafting, 8.7%; lobectomy, 7.8%; thyroidectomy, 12.7%; and pediatric inguinal hernia repair, 15.0%).^30^ In particular, the total numbers of pancreatic and liver surgeries in 2020 increased compared to those in 2019. One of the reasons might be that the timing of diagnosis of these tumors was not affected by the reduction of common cancer screenings caused by the pandemic. Another reason for the increase in pancreatic and liver surgeries might be that most of these procedures are indicated for malignant tumors, including pancreatic and biliary cancers, which are well‐recognized to show rapid growth and poor prognosis, prompting surgeons to perform surgeries without delay even in the COVID‐19 situation. On the other hand, the number of splenic surgeries, most of which are performed for benign diseases, decreased greatly in 2020 (13.1% decrease year‐on‐year). The surgical indications under the catastrophic situation of a pandemic need further investigations.

There are several trends for all gastroenterological surgeries in the last decade. The 30‐day mortalities were stable, and the operative mortalities were slightly improved for almost all digestive organs. Meanwhile, the incidence of postoperative complications in alimentary tract surgery, except the esophagus, increased slightly to 10% from 8% in these 10 years. Furthermore, in pancreas and esophagus surgery, these incidences showed a rapid increase (up to more than 20%). Otherwise, there were the following trends: surgical cases were consistently aging; participation of board‐certified surgeons increased progressively; and rates of endoscopic surgery increased. The year 2020 was the third year that robotic surgery procedures in the gastroenterological field became widely covered by insurance in Japan. The number of robotic surgeries is rapidly increasing,^31^, ^32^ and the statistics for these procedures were included as endoscopic surgeries in this report.

More than half of all patients who underwent surgical treatment in the gastroenterological field were aged 70 years or over in recent years. The rate of surgical cases for patients aged more than 70 years was 51.6% for all gastroenterological surgeries in 2020, although it was less than 40% in 2011. In particular, 2020 was the first year that the percentage of surgeries for patients aged 80 years or older was more than 20% for all gastroenterological surgeries (Table 3). However, the operative mortalities in this decade showed a decrease rather than an increase. There are several possible reasons for such favorable results and one of these might be a positive aspect of the increase of the participation rate of board‐certified surgeons in gastroenterological surgeries because the contribution of board‐certified surgeons to favorable outcomes was demonstrated in the major gastroenterological surgeries.^33^

The board‐certification system of the JSGS consists of board‐certified training institutions and board‐certified surgeons in gastroenterology.^33^ The rates of participation of JSGS board‐certified surgeons in alimentary tract surgery, except the esophagus, increased by approximately 15% compared to 10 years earlier. Moreover, more than 95% of esophagus or pancreas surgeries that had been performed in 2020 involved board‐certified surgeons. The decreases of operative mortalities and the paradoxical increases of postoperative complications in pancreas and esophagus surgery may be explained by the participation of board‐certified surgeons. The re‐operation rates of esophagectomy or pancreaticoduodenectomy stayed constant through the last 10 years. To avoid re‐operation or surgical death, invasive treatments for postoperative complications (grade IIIa of the C‐D classification) such as image‐guided percutaneous drainage might have been selected by board‐certified surgeons.

Many studies are in progress to improve the quality control of surgical procedures using the NCD. The JSGS approved 92 studies from 2013 to 2021 and many high‐impact articles that could contribute to the improvement of surgical quality have been published so far. Fifteen articles using the data of the NCD were published in 2021. For instance, the importance of board‐certified surgeons to reduce operative mortality was reported in hepatectomy and hepatopancreatoduodenectomy.^34^, ^35^ Additionally, hospital volume affected postoperative mortality after eight major gastroenterological surgeries.^36^

Regarding the studies of older patients in the NCD, the safety and feasibility in older rectal cancer patients were reported.^37^ Furthermore, with the aging society, the number of surgeries with ileostomy or colostomy has increased gradually in Japan.^22^ On the other hand, advanced age is associated with significantly worse short‐term outcomes in older patients undergoing major gastroenterological elective surgeries.^38^ However, any distinct cutoff age beyond which major gastroenterological surgery could be considered as being contraindicated was not identified.

In the investigation of minimally invasive surgery in the NCD, laparoscopic surgery was becoming common in surgery for acute diffuse peritonitis of gastrointestinal perforation in the data of the NCD.^39^ In the pancreatic field, the outcomes of laparoscopic pancreaticoduodenectomy under the strict limitations on institutions and indications were reported comparable to those of open procedures.^40^ Furthermore, robotic gastrectomy and low anterior resection in the NCD were safely performed, with low mortality and morbidity rates, either equaling or surpassing those of laparoscopic surgery.^31^, ^32^

With respect to the other issues of postoperative mortality and morbidity rates in the NCD, the impact of reconstruction route on postoperative morbidity after esophagectomy was reported.^41^ The Glasgow prognostic score was shown to be strongly correlated with postoperative outcomes.^42^ Moreover, the mortalities after right hemicolectomy or pancreatoduodenectomy were not affected by day of the week.^43^, ^44^ In addition, in emergency surgery for acute diffuse peritonitis, antithrombotic drug usage was associated with a slight increase of intraoperative blood loss, which was thought to have little effect on clinical practice.^45^

Much new evidence using the huge database of the NCD has been successively published. The NCD has contributed to quality assessment and improvement of surgery by feedback of accurate data in Japan. In conclusion, the good quality and safety of Japanese gastroenterological surgeries, which might be positively affected by the board‐certification system of the JSGS, were confirmed by the 10‐year data of the NCD. It is our hope that this report contributes to improving the quality of gastroenterological surgery in this country.

## CONFLICT OF INTEREST

Yuko Kitagawa is Editor in Chief of *Annals of Gastroenterological Surgery*. Hideki Ueno, Yoshihiro Kakeji, Susumu Eguchi, Akio Saiura, Hiroya Takeuchi, Naoki Hiki, Akihiko Horiguchi, Satoru Matsuda, Tsunekazu Mizushima, and Yasuyuki Seto are editorial board members of *Annals of Gastroenterological Surgery*. Arata Takahashi, Hiroyuki Yamamoto, and Hiroaki Miyata are affiliated with the Department of Healthcare Quality Assessment at the University of Tokyo. The department is a social collaboration department supported by grants from the National Clinical Database, Johnson & Johnson K.K., and Nipro Co and Intuitive Surgical Sàrl. Other authors have no conflict of interest.

## ETHICS STATEMENTS

Approval of the research protocol: N/A.

Informed Consent: N/A.

Registry and the Registration No. of the study/trial: N/A.

Animal Studies: N/A.
